# Assessment of evidence on reported non-genetic risk factors of congenital heart defects: the updated umbrella review

**DOI:** 10.1186/s12884-022-04600-7

**Published:** 2022-04-29

**Authors:** Xiaolu Nie, Xiaohang Liu, Chen Wang, Zehao Wu, Zimo Sun, Jian Su, Ruohua Yan, Yaguang Peng, Yuxuan Yang, Chengrong Wang, Siyu Cai, Yali Liu, Huanling Yu, Qingqing Wu, Xiaoxia Peng, Chenghong Yin

**Affiliations:** 1 Center for Clinical Epidemiology and Evidence-based Medicine, Beijing Children’s Hospital, Capital Medical University, National Center for Children Health, No.56 Nanlishi Road, Xicheng District, Beijing, 100045 China; 2 School of Public Health, Capital Medical University, Beijing, 100069 China; 3 Department of Scientific research, Beijing Obstetrics and Gynecology Hospital, Capital Medical University. Beijing Maternal and Child Health Care Hospital, Beijing, 100026 China; 4 Department of Nutrition and Food Hygiene, School of Public Health, Capital Medical University, Beijing, 100069 China; 5 Department of Ultrasound, Beijing Obstetrics and Gynecology Hospital, Capital Medical University. Beijing Maternal and Child Health Care Hospital, Beijing, 100026 China; 6 Department of Internal Medicine, Beijing Obstetrics and Gynecology Hospital, Capital Medical University. Beijing Maternal and Child Health Care Hospital, No.251 Yaojiayuan Road, Chaoyang District, Beijing, 100026 China

**Keywords:** Congenital heart defects, Non-genetic risk factors, Umbrella review, Grade of evidence

## Abstract

**Background:**

Congenital heart defect (CHD) is the leading cause of birth defects globally, which results in a great disease burden. It is still imperative to detect the risk factors of CHD. This umbrella review aimed to comprehensively summarize the evidence and grade the evidence of the associations between non-genetic risk factors and CHD.

**Methods:**

Databases including Medline, Embase, Web of Science, Cochrane Library, and four Chinese databases were searched from inception to 18 Jan 2022. The reference lists of systematic reviews (SR) and meta-analyses (MA) were screened, which aimed to explore the non-genetic risk factors of CHD. Subsequently, titles and abstracts of identified records and full texts of selected SR/MA were screened by two independent reviewers based on predefined eligibility criteria. A priori developed extraction form was used to abstract relative data following the PRISMA 2020 and MOOSE guidelines. The risk of bias was assessed with the AMSTAR2 instrument. Data were synthesized using fixed-effects and random-effects meta-analyses, respectively. Finally, the evidence on the association of non-genetic risk factors and CHD was graded using Ioannidis’s five-class evidence grade.

**Results:**

A total of 56 SRs, encompassing 369 MAs, were identified. The risk factors included relative factors on air pollution, reproductive-related factors, parental age and BMI, parental life habits, working and dwelling environment, maternal drug exposure, and maternal disease. Based on AMSTAR2 criteria, only 16% (9/56) of SRs were classified as “Moderate”. One hundred and two traceable positive association MAs involving 949 component individual studies were included in further analysis and grading of evidence. Family genetic history, number of abortions, maternal obesity, especially moderate or severe obesity, decoration materials, harmful chemicals, noise during pregnancy, folic acid supplementation, SSRIs, SNRIs, any antidepressants in the first trimester, maternal DM (including both PGDM and GDM), and gestational hypertension were convincing and highly suggestive factors for CHD. After sensitivity analyses based on cohort studies, some grades of evidence changed.

**Conclusion:**

The present umbrella review will provide evidence-based information for women of childbearing age before or during pregnancy to prevent CHD. In addition, sensitivity analysis based on cohort studies showed the changed evidence levels. Therefore, future SR/MA should concern the sensitivity analysis based on prospective birth cohort studies and case-control studies.

**Supplementary Information:**

The online version contains supplementary material available at 10.1186/s12884-022-04600-7.

## Introduction

Birth defects are growing parts of the global disease burden for under 18 years old because of the fall in infectious diseases and improvements in children nutrition, in which congenital heart defects (CHD) is the leading cause of birth defects globally [1]. More than one million fetuses with CHD worldwide result in a great disease burden [2], especially in less economically developed areas where treatment technologies for CHD are insufficient or unavailable [3]. Although the burden disease of CHD could be primarily controlled by prenatal screening for CHD, the increased rates of termination of pregnancy impacted the maternal psychological and physical health [4, 5]. Therefore, it is still imperative to reduce the risk of CHD and to enhance perinatal prevention and health care.

As for the risk of CHD, the current consensus is that the development of CHD is determined by both genetic and environment factors. Although the genetic algorithms for cardiac defects have been constructed, the risk assessment of CHD based on non-genetic risk factors is still imperative because non-genetic risk factors can be prevented more easily [6]. In order to clarify clear hierarchies of evidence between types of environmental factors and birth defect, especially for CHD, two umbrella reviews based on published systematic reviews and meta-analysis were performed [7, 8]. However, with the accumulation of new significant evidence on risk factors of CHD, including maternal diabetes mellitus (DM) [9], parental smoking [10], maternal air pollution exposure [11], maternal caffeinated products [12], and antidepressant classes and individual antidepressants [13], it was found that some associations of specific subgroup of CHD had not been contained and analyzed in the published reviews. In view of these developments, an updated umbrella review is needed to summary or evaluate the robustness of the evidence.

In addition, a China Birth Cohort aimed to assess the risk of CHD was initiated in 2017, 500,000 pregnant women have been enrolled and following up by far [14, 15]. In order to provide a comprehensive summary of non-genetic risk factors as a basis of this large cohort program, we designed this updated umbrella review to ascertain the validity and credibility of the published systematic reviews and meta-analyses for epidemiology studies on risk factors of CHD.

## Methods

This review was conducted according to the rules for conducting umbrella reviews and published approach [16, 17], and was reported in accordance with the Systematic Reviews and Meta-analysis (PRISMA 2020) statement [18] and Meta-analysis of Observational Studies in Epidemiology (MOOSE) guidelines [19].

### Literature search

The Chinese and English databases were systematically searched, including Medline, Embase, the Cochrane Library, Web of Science databases, Wangfang, CNKI, VIP, and Sinomed databases from database inception to 18 January 2022. All studies aimed to explore the potential environmental risk factors of CHD were captured. Initial free-text keywords and Medical Subject Headings or EmTree terms included ‘congenital heart defects’, ‘tetralogy of fallot’, ‘cyanotic heart’, ‘aortic coarctation’, ‘heart valve diseases’, ‘hypoplastic syndrome’, ‘pulmonary atresia’, ‘interruption of the aortic arch’, ‘valve stenosis’, ‘pulmonary atresia’, ‘systematic review’, and ‘meta-analysis’. To provide comparable results, we used the syntax applied in the previous comprehensive Cochrane reviews [20, 21] .The detailed search strategy can be found in Supplementary Table S1. All studies that included the search terms in the titles or abstracts were identified. To supplement the database searches, we further hand-searched the additional potential eligible studies according to the references of the published umbrella reviews as a supplementary search [7, 8].

### Eligibility criteria

The systematic reviews (SR) or meta-analyses (MA) of individual observational studies (case-control, cohort, cross-sectional and ecological studies) were eligible, which aimed to examine the associations between environmental risk or protective factors and CHD (including any kind of specific classification of CHD). The exclusion criteria included: (1) SR/MA focused only on genetic risk factors of CHD; (2) SR/MA focused on risk factors which influenced treatment and prognosis of CHD; (3) SR/MA aimed to study the impact of adult CHD on other diseases; (4) SR/MA of epidemiological descriptive studies of CHD; (5) SR/MA that did not present study specific data (relative risks (RR), odds ratio (OR), 95% confidence intervals, and numbers of cases/population). The language was restricted to English and Chinese. SR presented separate MA on more than one eligible outcome (such as atrial septal defect (ASD), ventricular septal defects (VSD), and coarctation of the aorta (COA)) were assessed separately. Given that more than one MA focus on the same scientific association, the one with the largest number of included component studies was selected, but sensitivity analyses and comparisons were conducted to assess the concordance of the summary associations (direction, magnitude, and significance) in these duplicate meta-analyses [22].

### Screening process and data extraction

Individual studies of SR and MA were firstly screened based on titles and abstracts. If a judgment could not be made based on titles and abstracts, we proceeded to read the full text. Both the screening process and data extraction were performed independently by four investigators (L.X., W.C., S.J., and S.Z.). Senior investigators (X.N.) resolved discrepancies through discussions.

For each eligible MA, three independent investigators (L.X., W.C., and S.J.) firstly extracted data including: name of first author, year of publication, country, factor, outcome, number of included component studies, search date, study population, combined effect value reported with 95% CI, the model of analysis (fix/random model), and method of bias assessment.

Four independent investigators (L.X., W.C., S.J., and S.Z.) then extract the following information for each component included study of eligible MA: first author and published year of corresponding MA, first name of component study, year of publication, study design, factor, outcome (including CHD and any kind of specific classification), comparison level, population size of each component study, number of case and control for case-control study, number of exposure and non-exposure group for cohort study, effect size reported with 95% CI. For the purpose of mitigating the risk of introducing newly defined factors not originally present in the literature, we restricted the data extraction to only the factors that each individual meta-analysis or systematic review had originally introduced and did not combine similar factors if the meta-analysis or systematic review had considered and analyzed them separately.

### Risk of bias assessment

The methodological quality of each included SR was independently assessed by two group of raters (L.X., W.C., S.J., and S.Z.) with the Assessment of Multiple SysTemAtic Reviews (AMSTAR) 2 tool (https://amstar.ca/Amstar-2.php). AMSTAR2 ranks the quality of a SR from critically low to high according to 16 predefined items [23]. In case of disagreement between raters, consensus ratings were used and senior investigator (X.N.) resolved discrepancies through discussions.

### Data synthesis and analysis

All statistical analyses and forest plot were conducted using the package ‘metaumbrella’ (version 1.0.1), which had just released in 2022 for R (R Foundation for Statistical Computing, version 4.1.2).

Considering the MA with negative association did not show statistical significance and the requirement of Egger test (number of included studies: k ≥ 3), we only focused on selected MA with positive association for further synthesis and analysis. For each eligible MA, we estimated the summary effect sizes and 95% CI through both fixed-effects and random-effects models. We also estimated the prediction interval (PI) and its 95%CI, which further accounts for between-study effects and estimates the certainty of the association if a new study addresses the same association [24–26]. Between-study inconsistency was estimated with the *I*^*2*^, with values > 50% indicative of high heterogeneity [27]. We calculated the evidence of small-study effects using the Egger test with a *p*-value of < 0.10 [28], where statistical significance would mean potential reporting/publication bias in smaller studies or other reasons why small studies differ from larger ones. Finally, we applied the excess of significance test [29]. Because of the limited statistical power of this test, a lenient significance threshold (*p* < 0.10) was adopted [30]. Considering the effect size of the largest dataset, we estimated the power of each component study with an algorithm using a non-central *t* distribution.

In addition, we addressed temporality with a sensitivity analysis that includes only prospective studies because the temporality of the association is critical to minimize reverse causation in an umbrella review of potential risk and protective factors [16, 31].

### Assessment of evidence credibility

All the evidences were categorized into five categories as follows: (1) Convincing: number of all included studies>1000 cases, random-effect *p*<10^− 6^, *I*^*2*^<50%, 95% prediction interval excluding null value, largest study has significantly result, no small study effect, no excess significance bias; (2) Highly suggestive: number of all included studies>1000 cases, random-effect *p*<10^− 6^, largest study has significantly result; (3) Suggestive: number of all included studies>1000 cases, random-effect *p*<10^− 3^; (4) Weak: significant association with *p*<0.05; (5) NS: Not significant associated [32].

## Results

### Characteristics of included SR/MA

**Fig. 1:**
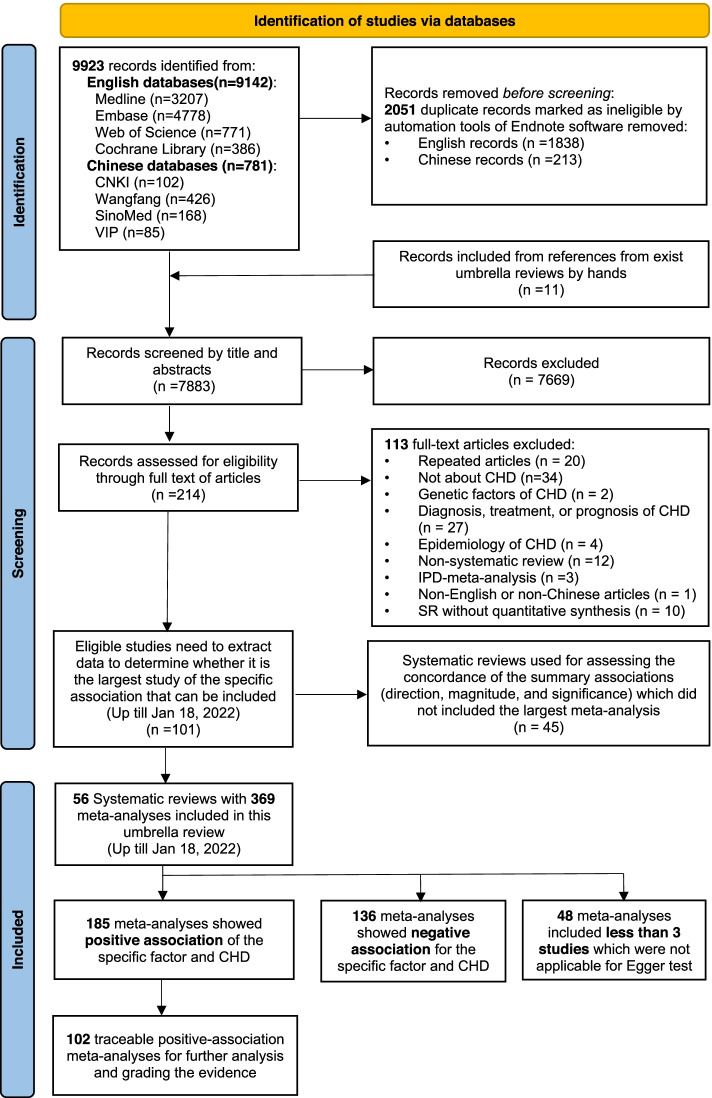
The flow diagram of included systematic reviews and meta-analysis

Overall, 9923 potentially eligible records were identified. After screening titles and abstracts, full-text evaluation was carried out for 214 records. Among them, 101 studies needed to extract data to determine whether it was the largest and latest study of the specific association that can be included. Finally, 56 SRs with 369 MAs were fulfilled the inclusion criteria (Fig. 1). In addition, the reference list of 45 excluded SR used for assessing the concordance of the summary associations is showed in Supplementary Table S2. The included studies examined a total of potential risk/protective factors in 6 categories including air pollution [11, 33–35], reproductive related factors [36–42], parental demographic status(i.e. age and BMI) [43–49], parental life habits, working and dwelling environment [10, 12, 42, 46, 50–55], maternal drug exposure [13, 53, 56–74], and maternal diseases [9, 75–82]. Fourteen multiple subtypes of CHD were involved atrial septal defect (ASD), ventricular septal defects (VSD), atrioventricular septal defect (AVSD), pulmonary valve stenosis (PVS), tetralogy of fallot (TOF), conotruncal defects (CTD), coarctation of the aorta (COA), patent ductus arteriosus (PDA), septal defects, transposition of great arteries (TGA), hypoplastic left heart syndrome (HLHS), outflow tract (OFT) defect, left ventricular outflow tract obstruction (LVOTD), and right ventricular outflow tract obstruction (RVOTD). Among all these 369 kinds of MAs for specific association, 50% (185/369) of MAs showed positive association for the specific factor and CHD, 37% (136/369) showed negative association, and 13% (48/369) included less than 3 studies which were not applicable for Egger test. Supplementary Table S3, S4, S5 shows the main characteristics of all the selected MAs. Given that negative association classified as lowest class and some data of specific positive-association MAs could not be traceable, we only focused on 102 traceable positive-association MAs for further analysis and grading the evidence (Fig. 1).

### Studies methodological quality and risk of bias assessment

The results of methodological quality ranking of all the included 56 SRs are shown in Supplementary Table S6. Based on AMSTAR2 criteria, 16% (9/56) of SRs were classified as “Moderate”, 29% (16/56) were “Low quality”, and 55% (31/56) were assessed as “Critically Low”. The critical flaws were mainly manifested in the following items: (1) All (0/56) SR did not report on the sources of funding for the individual studies; (2) 75% (42/56) did not provide a list of excluded studies to justify the exclusions; (3) 50% (28/56) did not assess the potential impact of risk of bias in individual studies on the results of the meta-analysis; (4) 45% (25/56) did not report the risk of bias in individual studies when interpreting/discussing the results of the review; (5) 41% (23/56) did not report any potential sources of conflict of interest.

### Overall data synthesis and analysis of eligible positive-association MAs

One-hundred and two MAs involving 949 component individual studies were included for data synthesis and grading the evidence, in which 271 individual studies were cohort studies and other 678 were case-control studies. Among them, the outcome of 802 studies were CHD, the rest were about ASD (*n* = 36), HLHS (*n* = 20), ASD/VSD (*n* = 17), septal defects (*n* = 16), TOF (*n* = 12), RVOTO (*n* = 10), COA (*n* = 7), CTD (*n* = 7), OFT defects (*n* = 6), VSD (*n* = 6), TGA (*n* = 4), and AVSD (*n* = 3). Table 1 and Table 2 showed the quantitative synthesis of eligible associations for specific factors and CHD together with various subtypes, respectively. Seventy-one of 102 (70%) associations were obtained from individual studies with 1000 or above cases. Forty-three (4%) associations had a *p* < 0.005, and 26 (25%) associations reached *p* < 10^− 6^. Large estimates of heterogeneity (*I*^*2*^ > 50%) in meta-analysis were detected for 38 association (37%). Moreover, small-study effects were showed in 19 associations (19%), and there were 24 associations (24%) with evidence of excess significance. Overall, 5 (5%) of all the factors showed convincing (Class I) evidence, 13 (13%) showed highly suggestive (Class II) evidence, 21 (21%) showed suggestive (Class III) evidence, 63 (62%) showed weak (Class IV) evidence. For presentation purposes, the sections below only summarized the evidence grade for 68 factors of CHD.

**Table 1:** Quantitative synthesis of eligible positive associations for specific risk/protective factor and CHD

Factor	Studies	Cases	Egger test	Random-effect	95%PI	Heterogeneity	Excess significance bias	Fixed-effect	Random-effect	Grade^**a**^
			***p*** value	Summary effect size(95%CI)		***I***^***2***^(%)	***p*** value	***p*** value	***p*** value	
**Reproductive related and assistive technologies**										
Family genetic history	5	7751	0.42	3.35 (2.70,4.14)	(2.37,4.73)	0.00	0.24	1.02E-28	1.02E-28	I
Abortion number	4	2413	0.45	1.28 (1.18,1.40)	(1.06,1.56)	55.24	0.34	1.89E-08	1.91E-08	II
Maternal parity	14	38,027	0.88	1.22 (1.09,1.36)	(0.83,1.78)	82.65	0.94	3.80E-45	4.22E-04	III
Singleton IVF/ICSI	5	2159	0.11	1.56 (1.21,2.00)	(0.79,3.08)	35.18	0.66	3.23E-05	5.89E-04	III
ICSI/IVF pregnancies	8	1047	0.61	1.45 (1.21,1.73)	(0.94,2.23)	43.62	0.57	5.78E-08	4.69E-05	III
History of spontaneous abortion	9	5377	0.54	1.21 (1.12,1.31)	(1.10,1.34)	11.15	0.64	2.57E-06	2.57E-06	III
History of abortion	13	7957	0.13	1.22 (1.11,1.34)	(1.00,1.48)	45.77	0.03	3.75E-07	2.49E-05	III
Gravidity number	7	4381	0.09	1.15 (1.08,1.22)	(0.99,1.33)	41.68	0.47	2.37E-08	7.12E-06	III
Maternal or fetal abnormalities detected	3	364	0.55	2.37 (1.25,4.49)	(0.02,296.12)	18.79	0.10	3.86E-03	7.88E-03	IV
Intermarriage	3	467	0.36	2.88 (1.88,4.39)	(0.18,44.83)	0.00	0.53	1.03E-06	1.03E-06	IV
ICSI vs IVF (in fresh transplantation cycle)	3	72	0.08	2.07 (1.28,3.36)	(0.09,47.47)	0.00	1.00	3.05E-03	3.05E-03	IV
History of induced abortion	6	1566	0.03	1.68 (1.10,2.55)	(0.44,6.41)	65.48	1.00	1.06E-03	1.64E-02	IV
Gravidity	10	5464	0.62	1.18 (1.03,1.36)	(0.77,1.81)	62.10	1.00	1.11E-05	2.03E-02	IV
MC twins without TTTS	5	134	0.77	5.44 (3.66,8.08)	(2.86,10.34)	0.00	0.94	5.57E-17	5.57E-17	IV
MC twins with TTTS	6	146	0.04	12.50 (8.66,18.04)	(7.43,21.03)	0.00	0.68	2.06E-41	2.06E-41	IV
MC twins	6	141	0.03	5.88 (4.18,8.28)	(3.62,9.55)	0.00	0.33	3.62E-24	3.62E-24	IV
MC twins with TTTS vs. MC twins without TTTS	4	123	0.67	2.40 (1.64,3.51)	(1.04,5.53)	0.00	0.74	6.62E-06	6.62E-06	IV
**Parental age and BMI**										
Maternal severe obesity	5	1497	0.10	1.38 (1.30,1.47)	(1.26,1.53)	0.00	0.25	1.99E-26	1.99E-26	I
Maternal moderate obesity	5	3835	0.16	1.15 (1.10,1.20)	(1.05,1.27)	33.84	0.24	1.37E-12	4.40E-10	I
Maternal obesity	20	58,926	0.01	1.33 (1.22,1.46)	(1.02,1.75)	61.64	0.06	5.28E-42	6.93E-10	II
Paternal age (≥40 years)	11	7456	0.30	1.71 (1.31,2.23)	(0.68,4.29)	94.33	0.00	1.46E-23	9.22E-05	III
Paternal age (35–39 years)	5	11,219	0.15	1.14 (1.06,1.22)	(0.98,1.32)	16.64	0.94	8.50E-06	1.72E-04	III
Advanced maternal age (≥35 years)	9	19,212	0.26	1.15 (1.07,1.24)	(0.98,1.36)	22.02	0.01	1.37E-06	2.66E-04	III
Maternal overweight	19	52,606	0.16	1.06 (1.01,1.12)	(0.93,1.21)	58.58	0.05	1.21E-04	2.20E-02	IV
**Parental life habits, working and dwelling environment**										
Exposure to noise during pregnancy	5	1218	0.03	2.80 (2.09,3.76)	(1.46,5.39)	31.76	0.10	3.28E-14	6.68E-12	II
Exposure to harmful chemicals during pregnancy	13	3300	0.76	3.35 (2.19,5.13)	(0.88,12.81)	63.80	0.01	2.34E-28	2.82E-08	II
Exposure of decoration materials during pregnancy	3	3090	0.46	4.21 (2.38,7.47)	(0.03,265.47)	74.50	0.55	6.37E-49	9.68E-07	II
Maternal educational attainment	30	27,642	0.29	1.13 (1.05,1.21)	(0.90,1.42)	57.14	0.39	4.95E-33	6.01E-04	III
Paternal smoking	10	8898	0.46	1.42 (1.17,1.73)	(0.72,2.82)	84.85	1.00	8.41E-17	4.07E-04	III
Paternal active smoking	13	2099	0.44	1.43 (1.19,1.72)	(0.85,2.42)	52.99	1.00	6.87E-12	1.18E-04	III
Maternal passive smoking	44	15,143	0.00	2.00 (1.65,2.43)	(0.61,6.55)	89.51	0.03	9.58E-55	2.09E-12	III
Maternal active smoking	85	123,755	0.00	1.30 (1.17,1.44)	(0.57,2.97)	88.20	0.18	3.39E-18	1.11E-06	III
Paternal occupational exposure to adverse substances	3	919	0.95	1.70 (1.19,2.43)	(0.17,17.30)	0.00	0.57	3.81E-03	3.81E-03	IV
Family income	5	8150	0.72	1.05 (1.01,1.10)	(0.98,1.13)	0.00	0.37	2.06E-02	2.06E-02	IV
Solvents exposure	6	2526	0.53	1.32 (1.06,1.63)	(0.97,1.78)	0.00	1.00	1.15E-02	1.15E-02	IV
Paternal heavy smoking (≥20cigarrette/day)	5	1813	0.03	1.85 (1.01,3.40)	(0.19,18.04)	85.41	0.00	1.22E-02	4.79E-02	IV
Paternal light smoking (10–19 cigarette/day)	4	1580	0.79	1.41 (1.13,1.76)	(0.64,3.11)	43.08	0.01	4.70E-05	2.48E-03	IV
High intake of caffeinated products	4	320	0.29	1.32 (1.09,1.60)	(0.86,2.02)	0.00	0.75	5.12E-03	5.12E-03	IV
Lithium exposure (in the first trimester compared with patients with bipolar disorderd)	3	59	0.39	1.96 (1.13,3.39)	(0.06,69.51)	0.00	1.00	1.69E-02	1.69E-02	IV
Lithium exposure (in the first trimester compared with general population)	4	15,293	0.41	4.90 (1.72,13.96)	(0.07,322.10)	69.14	0.08	2.70E-07	2.95E-03	IV
Lithium exposure (in the first trimester compared with unexposed women)	3	15,314	0.28	4.56 (1.59,13.12)	(0.00,627,861.00)	71.78	0.06	1.41E-06	4.89E-03	IV
**Maternal drug exposure**										
SNRIs	4	24,743	0.79	1.67 (1.40,1.98)	(1.09,2.55)	24.27	0.60	9.48E-10	6.07E-09	I
SSRIs	16	43,170	0.78	1.26 (1.19,1.33)	(1.19,1.34)	32.77	0.37	1.72E-16	1.73E-16	I
Folic acid supplementation	20	18,276	0.00	0.61 (0.51,0.73)	(0.30,1.22)	79.76	0.00	2.35E-21	2.14E-08	II
Any antidepressant (in the first trimester)	20	61,539	0.50	1.28 (1.17,1.41)	(0.98,1.69)	48.75	0.46	2.19E-22	2.07E-07	II
Fluoxetine	14	74,523	0.33	1.30 (1.13,1.50)	(0.98,1.72)	28.25	0.19	3.90E-06	2.40E-04	III
SSRIs (in the first trimester)	19	74,191	0.63	1.26 (1.13,1.42)	(0.86,1.87)	56.06	0.16	4.29E-15	8.23E-05	III
Oral hormone pregnancy tests	7	1003	0.76	1.90 (1.26,2.86)	(0.93,3.86)	0.00	0.95	5.75E-04	2.11E-03	IV
Sertraline	13	74,598	0.26	1.44 (1.10,1.91)	(0.59,3.53)	63.82	0.02	4.33E-06	9.22E-03	IV
Nitrate (each additional daily 0.5 mg)	3	826	0.56	1.02 (1.00,1.04)	(0.89,1.17)	0.00	0.15	4.26E-02	4.26E-02	IV
Citalopram	11	67,622	0.21	1.26 (1.05,1.50)	(0.82,1.94)	45.51	0.29	1.04E-03	1.12E-02	IV
Nitrate (high vs low)	4	912	0.14	1.20 (1.02,1.42)	(0.83,1.73)	0.00	1.00	3.28E-02	3.28E-02	IV
β-blockers (in the first trimester)	8	59,756	0.72	1.57 (1.11,2.23)	(0.59,4.18)	65.02	0.99	2.17E-06	1.08E-02	IV
Bupropion	3	6591	0.63	1.23 (1.01,1.49)	(0.35,4.31)	0.00	1.00	3.58E-02	3.58E-02	IV
Fluconazole (in the first trimester)	5	6716	0.44	1.95 (1.18,3.21)	(0.35,10.78)	78.00	0.11	1.18E-07	8.97E-03	IV
**Maternal diseases**										
Gestational hypertension	23	138,067	0.13	1.73 (1.48,2.03)	(0.85,3.51)	79.93	0.83	3.92E-128	1.19E-11	II
GDM	26	99,010	0.11	1.94 (1.59,2.35)	(0.82,4.57)	88.99	0.01	3.24E-114	3.82E-11	II
PGDM	30	139,743	0.04	3.13 (2.65,3.69)	(1.42,6.88)	79.39	0.79	0.00E+ 00	2.74E-41	II
DM	50	166,545	0.00	2.60 (2.65,3.01)	(1.09,6.24)	97.66	0.00	8.18E-61	1.69E-38	II
Fever	16	37,269	0.00	1.46 (1.21,1.76)	(0.75,2.85)	80.29	0.00	5.07E-05	7.76E-05	III
Chronic diseases before pregnancy	3	346	0.99	4.33 (2.28,8.23)	(0.07,227.22)	0.00	0.45	7.40E-06	7.40E-06	IV
Infection of the reproductive system	3	2742	0.01	4.57 (1.10,18.92)	(0.00,3000.00)	65.91	0.27	1.92E-03	3.61E-02	IV
Respiratory infection	5	653	0.28	3.79 (2.32,6.19)	(0.94,15.24)	57.54	0.69	1.26E-16	1.02E-07	IV
Malnutrition during pregnancy	4	538	0.14	1.96 (1.33,2.88)	(0.46,8.42)	48.51	0.01	2.51E-06	6.40E-04	IV
Influenza	8	6956	0.18	1.73 (1.10,2.71)	(0.59,5.03)	52.16	0.99	2.36E-06	1.73E-02	IV
Rubella virus	7	332	0.46	3.30 (2.36,4.62)	(2.13,5.12)	46.53	0.05	2.85E-12	2.86E-12	IV
Cytomegalovirus infection	4	119	0.42	3.95 (1.75,8.90)	(0.53,29.38)	53.34	0.33	3.28E-04	9.43E-04	IV
Viral infection	17	6401	0.15	2.53 (1.40,4.56)	(0.23,27.47)	77.67	0.38	1.91E-15	2.15E-03	IV

^a^ Ioannidis’s five-class evidence grade

*Abbreviation*: *CI* Confidence interval, *PI* Predictive interval, *IVF* In-vitro-fertilization, *ICSI* Intracytoplasmic sperm injection, *SSRI* Selective serotonin reuptake inhibitor, *SNRI* serotonin-norepinephrine reuptake inhibitor, *DM* maternal diabetes mellitus, *PGDM* pregestational diabetes mellitus, *GDM* gestational diabetes mellitus, *TTTS* twin–twin transfusion syndrome, *MC* Monochorionic

**Table 2:** Quantitative synthesis of eligible positive associations for specific factor and subtypes of CHD

Factor	Studies	Cases	Egger test	Random-effect	95%PI	heterogeneity	excess significance bias	Fixed-effect	Random-effect	Grade^**a**^
			***p*** value	summary effect size(95%CI)		***I***^***2***^(%)	***p*** value	***p*** value	***p*** value	
**ASD**										
SSRIs (in the first trimester)	6	6608	0.79	2.06 (1.40,3.02)	(0.68,6.23)	57.77	0.97	6.33E-10	2.21E-04	III
Maternal obesity	4	2328	0.27	1.38 (1.21,1.59)	(0.87,2.19)	28.15	0.71	5.98E-10	2.98E-06	III
Fever	6	2141	0.53	1.43 (1.01,2.39)	(0.37,5.55)	40.76	0.94	4.41E-02	3.00E-03	IV
SSRIs	7	2967	0.50	1.82 (1.24,2.68)	(0.56,5.92)	71.98	0.99	8.52E-08	2.37E-03	IV
Maternal active smoking	9	52,077	0.71	1.26 (1.02,1.61)	(0.59,2.72)	73.23	0.03	4.19E-09	3.93E-02	IV
Maternal severe obesity	3	90	0.39	1.72 (1.35,2.19)	(0.15,19.48)	46.79	0.00	3.35E-09	1.12E-05	IV
Maternal moderate obesity	3	346	0.45	1.26 (1.14,1.41)	(0.63,2.52)	0.00	0.07	1.79E-05	1.79E-05	IV
**HLHS**										
Nitrofurantoin (in the first trimester)	3	2845	0.22	3.07 (1.59,5.93)	(0.04,217.94)	0.00	0.73	8.25E-04	8.25E-04	III
Ondansetro	3	28,777	0.72	1.49 (1.03,2.17)	(0.13,16.79)	0.00	1.00	3.48E-02	3.48E-02	IV
Maternal obesity	4	146	0.17	1.52 (1.23,1.88)	(0.96,2.42)	0.00	0.42	9.15E-05	9.15E-05	IV
Maternal severe obesity	3	32	0.22	1.60 (1.11,2.31)	(0.15,17.43)	0.00	1.00	1.26E-02	1.26E-02	IV
Maternal moderate obesity	3	92	0.28	1.54 (1.21,1.95)	(0.33,7.11)	0.00	0.18	3.51E-04	3.51E-04	IV
Maternal overweight	4	148	0.00	1.31 (1.08,1.60)	(0.85,2.03)	0.00	1.00	7.18E-03	7.18E-03	IV
**ASD/VSD**										
SSRIs (in the first trimester)	16	31,414	0.15	1.29 (1.14,1.45)	(0.91,1.81)	41.86	0.18	1.08E-09	3.62E-05	III
**Septal defects**										
Sertraline	3	1428	0.32	3.17 (2.11,4.76)	(0.23,43.91)	0.00	0.36	2.40E-08	2.40E-08	II
SSRIs	6	7722	0.62	1.38 (1.02,1.86)	(0.56,3.42)	67.14	0.98	4.76E-05	3.46E-02	IV
Fluconazole (in the first trimester)	3	42,838	0.22	1.43 (1.06,1.93)	(0.10,19.66)	0.00	0.14	3.57E-03	1.98E-02	IV
Maternal obesity	4	3483	0.09	1.28 (1.03,1.59)	(0.63,2.59)	9.27	0.74	8.66E-05	2.91E-02	IV
**TOF**										
GDM	4	1696	0.60	1.51 (1.10,2.05)	(0.76,2.98)	0.00	0.33	9.92E-03	9.92E-03	IV
Maternal obesity	5	887	0.48	1.28 (1.09,1.51)	(0.99,1.66)	10.44	0.05	2.11E-03	2.11E-03	IV
Maternal severe obesity	3	648	0.14	1.95 (1.50,2.52)	(0.36,10.44)	0.00	0.59	4.54E-07	4.54E-07	IV
**RVOTO**										
Fever	3	2020	0.31	1.66 (1.04,2.63)	(0.01,229.96)	60.41	0.06	2.64E-03	3.22E-02	IV
SSRIs	4	4318	0.34	1.39 (1.09,1.77)	(0.61,3.16)	32.18	0.94	5.80E-04	8.45E-03	IV
Maternal active smoking	3	30,993	0.14	1.43 (1.03,1.99)	(0.07,28.91)	24.71	0.89	6.03E-03	3.11E-02	IV
**COA**										
Maternal obesity	4	534	0.36	1.25 (1.02,1.52)	(0.80,1.94)	0.00	1.00	3.02E-02	3.02E-02	IV
Maternal moderate obesity	3	502	0.62	1.29 (1.03,1.60)	(0.31,5.36)	0.00	1.00	2.42E-02	2.42E-02	IV
**CTD**										
Fever	4	2560	0.54	1.40 (1.01,2.02)	(0.47,4.18)	5.41	0.96	4.26E-02	3.68E-02	IV
Maternal obesity	3	1278	0.12	1.23 (1.08,1.40)	(0.52,2.88)	0.00	0.67	2.37E-03	2.37E-03	IV
**OFT**										
Maternal obesity	3	620	0.79	1.39 (1.25,1.55)	(0.62,3.15)	0.00	0.62	2.66E-11	8.89E-10	IV
Maternal overweight	3	998	0.14	1.19 (1.09,1.31)	(0.57,2.78)	0.00	0.17	1.50E-4	1.50E-4	IV
**TGA**										
Fever	4	1016	0.43	1.85 (1.11,3.09)	(0.48,7.12)	0.00	0.96	1.22E-02	1.85E-02	IV
**VSD**										
Gestational hypertension	4	32,978	0.00	1.29 (1.17,1.43)	(1.03,1.61)	58.06	0.03	7.84E-07	7.84E-07	II
Maternal severe obesity	3	424	0.41	1.09 (1.02,1.69)	(0.04,30.31)	14.99	1.00	7.04E-01	3.92E-02	IV
**AVSD**										
Maternal severe obesity	3	756	0.37	1.44 (1.03,2.00)	(0.17,12.39)	0.00	1.00	3.23E-02	3.23E-02	IV

^a^Ioannidis’s five-class evidence grade

*Abbreviation*: *CI* Confidence interval, *PI* Predictive interval, *IVF* In-vitro-fertilization, *ICSI* Intracytoplasmic sperm injection, *SSRI* Selective serotonin reuptake inhibitor, *SNRI* serotonin-norepinephrine reuptake inhibitor, *DM* maternal diabetes mellitus, *PGDM* pregestational diabetes mellitus, *GDM* gestational diabetes mellitus, *TTTS* twin–twin transfusion syndrome, *MC* Monochorionic, *ASD* Atrial septal defect, *HLHS* Hypoplastic left heart syndrome, *VSD* Ventricular septal defects, *TOF* Tetralogy of fallot, *RVOTD* Right ventricular outflow tract obstruction, *COA* Coarctation of the aorta, *CTD* Conotruncal defects, *OFT* outflow tract, *TGA* Transposition of great arteries, *AVSD* Atrioventricular septal defect

#### Reproductive related and assistive technologies

**Fig. 2:**
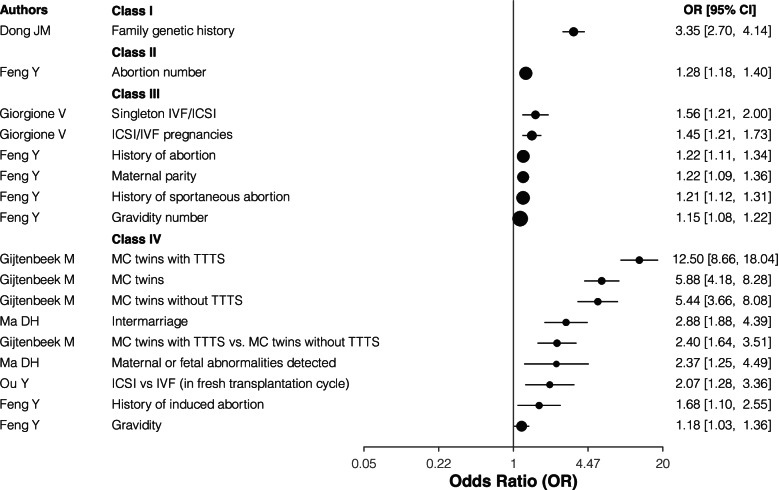
The forest plot for the association reproductive related and assistive technologies risk factors and CHD. IVF: In-vitro-fertilization; ICSI: Intracytoplasmic sperm injection; TTTS, twin–twin transfusion syndrome; MC, Monochorionic

Regarding the outcome of CHD, family genetic history showed convincing (Class I), and number of abortions were highly suggestive (Class II) evidence. In-vitro-fertilization (IVF) or Intracytoplasmic sperm injection (ICSI) pregnancy, especially singleton IVF/ICSI, history of (spontaneous) abortion, maternal parity, gravidity number, these five factors showed suggestive (Class III) evidence of increasing the risk for CHD. Monochorionic (MC) twins, either with or without twin–twin transfusion syndrome (TTTS), MC twins with TTTS vs. MC twins without TTTS, ICSI vs IVF (in fresh transplantation cycle), intermarriage, maternal or fetal abnormalities detected, history of induced abortion, and gravidity had class IV evidence as a risk factor for CHD (Table 1 and Fig. 2).

#### Parental age and BMI

**Fig. 3:**
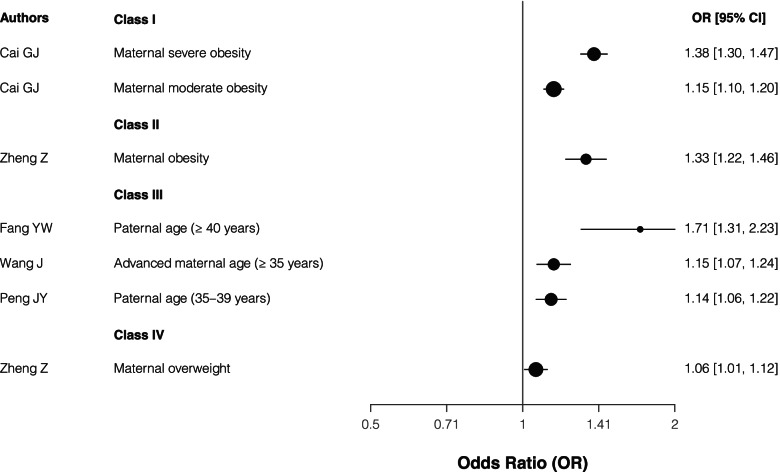
The forest plot for the association between parental age and BMI risk factors and CHD

Regarding the maternal BMI, maternal moderate or severe obesity identified convincing (Class I) evidence and maternal obesity showed highly suggestive (Class II) compared with normal BMI, while maternal overweight had class IV evidence as a risk factor for CHD. In terms of parental age, paternal age (≥40 years and 35–39 years) and maternal age (≥35 years) showed suggestive (Class III) evidence of increasing the risk for CHD (Table 1 and Fig. 3).

#### Parental life habits, working and dwelling environment

**Fig. 4:**
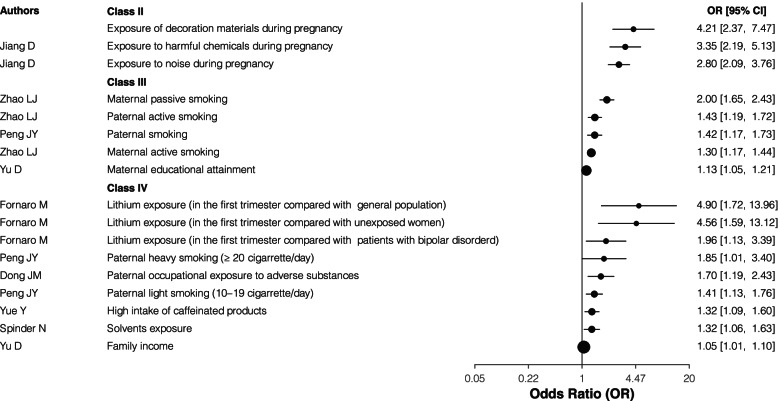
The forest plot for the association between parental life habits, working and dwelling environment and CHD

Exposure to decoration materials, harmful chemicals and noise during pregnancy showed highly suggestive evidence (Class II). While the weak (Class IV) evidence included the Lithium exposure in the first trimester compared with unexposed women or history exposed patients with bipolar disorder, solvents exposure, paternal occupational exposure to adverse substances, and high intake of caffeinated products. Regarding the parental smoking, both maternal active and passive smoking, paternal smoking, especially paternal active smoking were classed as grade III evidence. However, both paternal light smoking (10–19 cigarettes/day) and heavy smoking (≥20 cigarettes/day) were showed as weak (Class IV) evidence compared with nonsmoker. In terms of family financial situation, maternal educational attainment and family income showed suggestive (Class III) evidence and weak (Class IV) evidence, respectively (Table 1 and Fig. 4).

#### Maternal drug exposure

**Fig. 5:**
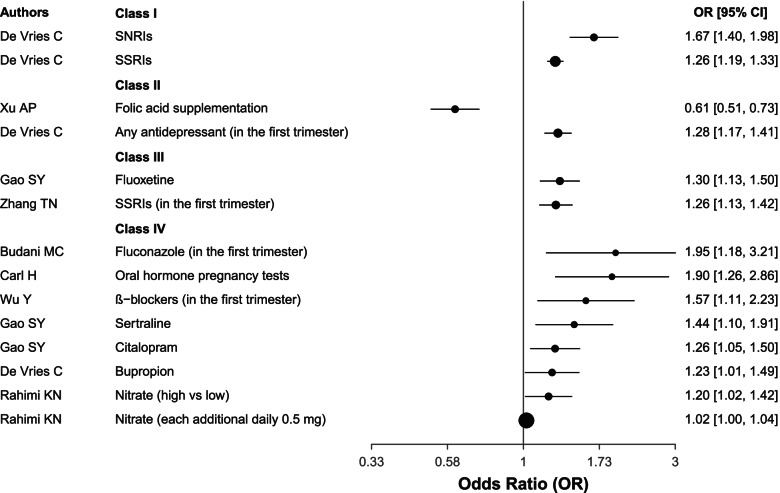
The forest plot for the association between maternal drug exposure and CHD. SSRI, Selective serotonin reuptake inhibitor; SSRI, Selective serotonin reuptake inhibitor

Folic acid supplementation showed convincing (Class II) evidence, which was only protective factor for CHD. On the contrary, Selective serotonin reuptake inhibitors (SSRIs) and serotonin-norepinephrine reuptake inhibitors (SNRIs) showed convincing (Class I) evidence as risk factors for CHD. Any antidepressants in the first trimester were class as grade II. Other eligible drug exposure, including fluconazole in the first trimester, ß − blockers in the first trimester, oral hormone pregnancy tests, sertraline, citalopram, bupropion, nitrate (either high vs low or each additional daily 0.5 mg) were class IV risk factors for CHD (Table 1 and Fig. 5).

#### Maternal diseases

**Fig. 6:**
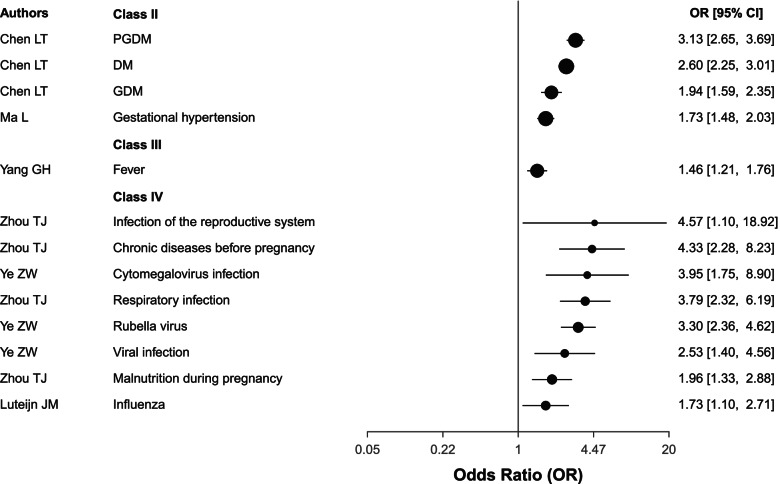
The forest plot for the association between maternal disease and CHD. PGDM, pregestational diabetes mellitus; DM, diabetes mellitus; GDM: gestational diabetes mellitus

Maternal diabetes mellitus (DM), including both pregestational diabetes mellitus (PGDM), gestational diabetes mellitus (GDM), together with gestational hypertension were classed as highly suggestive evidence (Class II). Fever showed suggestive (Class III) evidence. Finally, malnutrition during pregnancy, infection of the reproductive system, cytomegalovirus, rubella virus, influenza, viral infection, respiratory infection all had class IV evidence. Chronic disease before pregnancy was also a class IV risk factor for CHD (Table 1 and Fig. 6).

### Sensitivity analysis investigating temporality of association

The cohort studies were separated for sensitivity analyses involving 37 factors, in which seven factors (high intake of caffeinated products, solvents exposure, family income, folic acid supplementary, fluconazole in the first trimester, bupropion, and fever) did not conduct data quantitative synthesis because of only one eligible cohort (Supplementary Table S7 and Fig. S1, S2, S3, S4, S5). Among the other 30 factors, 24 remained significant at *p* < 0.05. Overall, 16 factors remained the same level of evidence with umbrella review based on both cohort studies and case-control studies. In addition, 13 factors (maternal parity, ICSI/IVF pregnancies, maternal obesity, maternal overweigh, paternal age (≥40 years), maternal educational attainment, maternal passive smoking, maternal active smoking, β-blockers in the first trimester, SNRI, SSRI, oral hormone pregnancy tests, and GDM) downgraded while one (any antidepressant in the first trimester) upgraded.

## Discussion

We conducted this updated umbrella review to systematically integrate the evidence to data of risk/protective factors for CHD and its various subtypes. In summary, our umbrella review indicated that family genetic history, number of abortions, maternal obesity, especially moderate or severe obesity, decoration materials, harmful chemicals, noise during pregnancy, folic acid supplementation, SSRIs, SNRIs, any antidepressants in the first trimester, maternal DM (including both PGDM and GDM), and gestational hypertension were convincing and highly suggestive factors for CHD.

Although there have been published two umbrella reviews, Zhang’s study lacked of not only some important factors, maternal DM for instance, but also assessment of robustness based on sensitivity analysis [7]; while Lee’s review focused on both environmental and genetic risk factors of all kinds of congenital anomalies rather than only about CHD which seem unreasonable because different types of congenital anomalies occurred based on different pathogenesis [8]. Furthermore, the evidence was not graded in Lee’s review. Given these aforementioned limitations, we searched till 18 Jan, 2022 and included all the latest SR/MA of specific association of CHD, such as maternal DM [9], parental smoking [10], and air pollution exposure [11], which are important factors for CHD and its various subtypes. Moreover, we conducted sensitivity analysis based on only cohort component individual studies to detect the robustness of current evidence based on both cohort studies and case-control studies. In addition, we used the latest released R package ‘metaumbrella’ (version 1.0.1) to conduct and check all the analysis process as recommended by the rules for conducting umbrella review [16], which facilitated quality control for process and better comparison of results.

Compared with the results of published umbrella reviews, most of the summary results are consistent and grade of factors stay the same. Regarding the inconsistent results, partly because we chose latest published largest MA for specific association, in which the component individual studies were different from articles of Zhang’s and Korean research groups [7, 8]. On the other hand, since some included MA did not provide the reference list or the complete data for analysis in this umbrella review so that we could not confirm the accuracy of the data in SR after careful consideration, even though we attempted to contact the corresponding author. Therefore, we waived to synthesis and analysis these associations and only summarized the main characteristics in Supplementary Table S4. It is suggested that meta-analysis should not only focus on reporting quality, but also provide necessary required data for subsequent repeatable analysis.

Our results suggest a substantial number of factors that may be considered as predictors in CHD (although their causality may be less certain and need further high -quality cohort research). The most obvious advantage of studying risk factors, particularly those that are environmental and potentially modifiable, is that it can provide crucial knowledge on prevention strategies [3]. The convincing and highly suggestive factors defined in our umbrella review, including family genetic history, number of abortions, maternal obesity, especially moderate or severe obesity, decoration materials, harmful chemicals, noise during pregnancy, folic acid supplementation, SSRIs, SNRIs, any antidepressants in the first trimester, maternal DM (including both PGDM and GDM), and gestational hypertension, should be focused by women of childbearing age before or during pregnancy to prevent fetal congenital heart disease.

The main strength of this umbrella review lies in the systematic search strategy, good quality control during data extraction, and rigorous data analysis and synthesis. However, this review does have some limitations. Firstly, as we described in the methods, this umbrella review could only conduct secondary analysis based on the associations which has been investigated, published and systematically reviewed or meta-analyzed. Take maternal MC twins with TTTS as an example, since there was only one MA (including two component individual studies) in Gijtenbeek’s article focused on this association [38], even if this association may have an amazingly strong effect, but it will probably only be classified as Class IV evidence because of involving < 1000 patients. To avoid this limitation, we systematically searched and included as comprehensive as possible. Indeed, if the factor was not part of any systematic review or meta-analysis, it would not be even included in the umbrella review. Moreover, since the overall quality of the included SRs and MAs was relatively unsatisfactory and data tracing could not be conducted, some factors could not be graded in our umbrella review (see in the Supplementary Table S4 for data traceablity). At last, relatively a few prospective cohort individual studies were included in current SR/MA so that further causality inference needs to be very cautious. Future research about CHD should be focused on establish larger birth cohort and continuously followed-up to provide more powerful sequential evidence.

## Conclusion

The present umbrella review will provide evidence-based information for women of childbearing age before or during pregnancy to prevent CHD. In addition, the sensitivity analysis based on cohort studies showed the changed evidence levels. Therefore, future SR/MA should concern the sensitivity analysis based on prospective birth cohort studies and case-control studies.

## Supplementary Information

**Additional file 1 **: Supplementary materials.

## Data Availability

The datasets analyzed during the current study are available from the corresponding author on reasonable request. All data were extracted from published systematic reviews and meta-analyses.

## Abbreviations

CHD: Congenital heart defects
IVF: In-vitro-fertilization
ICSI: Intracytoplasmic sperm injection
SSRI: Selective serotonin reuptake inhibitor
SNRI: Serotonin-norepinephrine reuptake inhibitor
DM: Diabetes mellitus
PGDM: Pregestational diabetes mellitus
GDM: Gestational diabetes mellitus
TTTS: Twin–twin transfusion syndrome
MC: Monochorionic
ASD: Atrial septal defect
HLHS: Hypoplastic left heart syndrome
VSD: Ventricular septal defects
TOF: Tetralogy of fallot
RVOTD: Right ventricular outflow tract obstruction
COA: Coarctation of the aorta
CTD: Conotruncal defects
OFT: Outflow tract
TGA: Transposition of great arteries
AVSD: Atrioventricular septal defect
