# Supplementary material for: Assessment of evidence on reported non-genetic risk factors of congenital heart defects: the updated umbrella review
Source: BMC Pregnancy Childbirth. 2022 Apr 29;22:371. doi: 10.1186/s12884-022-04600-7 (PMC9055777; doi:10.1186/s12884-022-04600-7)
Supplement: Supplementary file 1 — Additional file 1 : Supplementary materials. [file 12884_2022_4600_MOESM1_ESM.docx]

**Assessment of evidence on reported non-genetic risk factors of congenital heart defects: the updated umbrella review**

**Supplementary Materials**

**Supplementary Table S1** Search strategy

**Supplementary Table S2** The systematic reviews used for assessing the concordance of the associations which did not include the largest meta-analysis (n=45)

**Supplementary Table S3** The characteristic of specific positive association between environment risk factors and CHD (n=185)

**Supplementary Table S4** The characteristic of specific negative association between environment risk factors and CHD (n=136)

**Supplementary Table S5** The characteristic of specific association which the number of included studies less than three (n=48)

**Supplementary Table S6** AMSTAR 2 quality assessment of 56 included systematic reviews

**Supplementary Table S7** Sensitivity analysis of eligible associations for specific factor and CHD (only included cohort studies)

**Supplementary Figure S1** The forest plot of sensitivity analysis for the association between reproductive related and assistive technologies risk factors and CHD

**Supplementary Figure S2** The forest plot of sensitivity analysis for the association between parental age and BMI risk factors and CHD

**Supplementary Figure S3** The forest plot of sensitivity analysis for the association between parental life habits, working and dwelling environment risk factors and CHD

**Supplementary Figure S4** The forest plot of sensitivity analysis for the association between maternal drug exposure risk factors and CHD

**Supplementary Figure S5** The forest plot of sensitivity analysis for the association between maternal disease risk factors and CHD

**Supplementary Table S1 Search strategy**

**Embase**

| **Serial number** | **Search terms** | **Number of articles** |
| --- | --- | --- |
| [#1](https://www.ncbi.nlm.nih.gov/pubmed) | 'congenital heart malformation'/exp | 161,378 |
| [#2](https://www.ncbi.nlm.nih.gov/pubmed) | 'valvular heart disease'/exp | 204,366 |
| [#3](https://www.ncbi.nlm.nih.gov/pubmed) | 'congenital heart malformation' | 41,817 |
| [#4](https://www.ncbi.nlm.nih.gov/pubmed) | tetralogy NEAR/3 fallot* | 20,906 |
| [#5](https://www.ncbi.nlm.nih.gov/pubmed) | cyanotic NEAR/3 heart | 4,392 |
| [#6](https://www.ncbi.nlm.nih.gov/pubmed) | congenital NEAR/3 cardiac* | 10,996 |
| [#7](https://www.ncbi.nlm.nih.gov/pubmed) | aortic NEAR/3 coarctation | 16,454 |
| [#8](https://www.ncbi.nlm.nih.gov/pubmed) | valve NEAR/3 disease* | 35,621 |
| [#9](https://www.ncbi.nlm.nih.gov/pubmed) | hypoplastic NEAR/3 syndrome | 7,727 |
| [#10](https://www.ncbi.nlm.nih.gov/pubmed) | pulmonary NEAR/3 atresia | 7,548 |
| [#11](https://www.ncbi.nlm.nih.gov/pubmed) | 'interruption of the aortic arch' | 371 |
| [#12](https://www.ncbi.nlm.nih.gov/pubmed) | valve NEAR/3 stenosis | 55,844 |
| [#13](https://www.ncbi.nlm.nih.gov/pubmed) | #1 OR #2 OR #3 OR #4 OR #5 OR #6 OR #7 OR #8 OR #10 OR #11 OR #12 | 354,370 |
| [#14](https://www.ncbi.nlm.nih.gov/pubmed) | 'systematic review'/exp | 327,481 |
| [#15](https://www.ncbi.nlm.nih.gov/pubmed) | 'meta analysis'/exp | 234,113 |
| [#16](https://www.ncbi.nlm.nih.gov/pubmed) | 'systematic review' | 414,906 |
| [#17](https://www.ncbi.nlm.nih.gov/pubmed) | 'meta analysis' | 343,597 |
| [#18](https://www.ncbi.nlm.nih.gov/pubmed) | #14 OR #15 OR #16 OR #17 | 570,814 |
| [**#19**](https://www.ncbi.nlm.nih.gov/pubmed) | **#13 AND #18** | **4778** |

**Cochrane Library**

#1 MeSH descriptor: [Heart Defects, Congenital] explode all trees

#2 MeSH descriptor: [Heart Valve Diseases] explode all trees

#3 (Congenital Heart Defects OR Heart Valve Diseases OR tetralogy near fallot* OR cyanotic near heart OR congenital near heart OR congenital near cardiac OR aortic near coarctation OR valve near diseases OR hypoplastic near syndrome OR pulmonary near atresia OR interruption of the aortic arch OR valve near stenosis OR pulmonary near atresia)

#1 or #2 or #3 limit in Cochrane Reviews  **386**

**Web of Science**

TS=congenital heart defect* AND（TS=systematic review OR TS=meta-analysis）  **771**

**Medline (PubMed)**

| **Serial number** | **Search terms** | **Number of articles** |
| --- | --- | --- |
| [#1](https://www.ncbi.nlm.nih.gov/pubmed) | (Heart Defects, Congenital[MeSH] OR Heart Valve Diseases[MeSH] OR tetralogy fallot* OR cyanotic heart OR congenital heart OR congenital cardiac OR aortic coarctation OR valve diseases OR hypoplastic syndrome OR pulmonary atresia OR interruption of the aortic arch OR valve stenosis OR pulmonary atresia) Sort by: Publication Date | 342,676 |
| [#2](https://www.ncbi.nlm.nih.gov/pubmed) | Search: "Heart Diseases/congenital"[Mesh] Sort by: Most Recent | 6,786 |
| [#3](https://www.ncbi.nlm.nih.gov/pubmed) | Search: #8 or #9 Sort by: Publication Date | 342,861 |
| [#4](https://www.ncbi.nlm.nih.gov/pubmed) | ("Meta-Analysis" [Publication Type]) OR （ "Systematic Review" [Publication Type]) OR (Meta analysis) OR (Meta analyses) OR (Systematic review) OR (Systematic reviews) Sort by: Publication Date | 385,437 |
| [**#5**](https://www.ncbi.nlm.nih.gov/pubmed) | **Search: #3 and #4 Sort by: Publication Date** | **3,207** |

**Sinomed (Chinese database)**

| **Serial number** | **Search terms** | **Number of articles** |
| --- | --- | --- |
| [#1](https://www.ncbi.nlm.nih.gov/pubmed) | 先天性心脏病 | [22,644](javascript:historyLink('先天性心脏病')) |
| [#2](https://www.ncbi.nlm.nih.gov/pubmed) | 先天性心脏缺损 | [98](javascript:historyLink('先天性心脏缺损')) |
| [#3](https://www.ncbi.nlm.nih.gov/pubmed) | 心脏瓣膜疾病/外科学 | 4,349 |
| [#4](https://www.ncbi.nlm.nih.gov/pubmed) | 法洛四联症 | [2,288](javascript:historyLink('法洛四联症')) |
| [#5](https://www.ncbi.nlm.nih.gov/pubmed) | 动脉导管未闭 | [18,987](javascript:historyLink('动脉导管未闭')) |
| [#6](https://www.ncbi.nlm.nih.gov/pubmed) | 房间隔缺损 | [27,937](javascript:historyLink('房间隔缺损')) |
| [#7](https://www.ncbi.nlm.nih.gov/pubmed) | 室间隔缺损 | [32,119](javascript:historyLink('室间隔缺损')) |
| [#8](https://www.ncbi.nlm.nih.gov/pubmed) | 肺动脉闭锁 | [754](javascript:historyLink('肺动脉闭锁')) |
| [#9](https://www.ncbi.nlm.nih.gov/pubmed) | 瓣膜狭窄 | [209](javascript:historyLink('瓣膜狭窄')) |
| [#10](https://www.ncbi.nlm.nih.gov/pubmed) | #1 OR #2 OR #3 OR #4 OR #5 OR #6 OR #7 OR #8 OR #9 | [92,424](javascript:historyLink('(瓣膜狭窄)%20OR%20(肺动脉闭锁)%20OR%20(室间隔缺损)%20OR%20(房间隔缺损)%20OR%20(动脉导管未闭)%20OR%20(法洛四联症)%20OR%20(心脏瓣膜疾病/外科学)%20OR%20(先天性心脏缺损)%20OR%20(先天性心脏病)')) |
| [#11](https://www.ncbi.nlm.nih.gov/pubmed) | 系统综述 or meta分析 | [222,137](javascript:historyLink('系统综述%20%20or%20%20meta分析')) |
| [#12](https://www.ncbi.nlm.nih.gov/pubmed) | #10 and #11 | [428](javascript:historyLink('(系统综述%20%20or%20%20meta分析)%20AND%20((瓣膜狭窄)%20OR%20(肺动脉闭锁)%20OR%20(室间隔缺损)%20OR%20(房间隔缺损)%20OR%20(动脉导管未闭)%20OR%20(法洛四联症)%20OR%20(心脏瓣膜疾病/外科学)%20OR%20(先天性心脏缺损)%20OR%20(先天性心脏病))')) |

**CNKI (Chinese database)**

(SU='先天性心脏病'+'先心病'+'先天性心脏缺损'+'心脏瓣膜疾病'+'法洛四联症'+'动脉导管未闭'+'房间隔缺损'+'室间隔缺损'+'肺动脉闭锁'+'瓣膜狭窄') AND (SU='系统综述'+'meta 分析')  **102**

**Wangfang (Chinese database)**

（主题=(先天性心脏病 OR 先心病 OR 先天性心脏缺损 OR 心脏瓣膜疾病 OR 法洛四联症 OR 动脉导管未闭 OR 房间隔缺损 OR 室间隔缺损 OR 肺动脉闭锁 OR 瓣膜狭窄) AND (主题=(系统综述 OR Meta分析 OR meta分析))

**426**

**VIP (Chinese database)**

题名或关键词=先天性心脏病 或者 题名或关键词=先心病 或者 题名或关键词=先天性心脏缺损 或者 题名或关键词=心脏瓣膜疾病 或者 题名或关键词=法洛四联症 或者 题名或关键词=动脉导管未闭 或者 题名或关键词=房间隔缺损 或者 题名或关键词=室间隔缺损 或者 题名或关键词=肺动脉闭锁 或者 题名或关键词=瓣膜狭窄 并且 题名或关键词=系统综述 或者 题名或关键词=meta分析 或者 题名或关键词=meta分析 **85**

**Supplementary Table S2 The systematic reviews used for assessing the concordance of the associations which did not include the largest meta-analysis (n=45)**

**ID Record**

1. Browne ML: Maternal exposure to caffeine and risk of congenital anomalies: a systematic review. *Epidemiology* 2006:324-331.

2. Rahimi R, Nikfar S, Abdollahi M: Pregnancy outcomes following exposure to serotonin reuptake inhibitors: a meta-analysis of clinical trials. *Reproductive Toxicology* 2006, 22(4):571-575.

3. Ge GM, Leung MTY, Man KKC, Leung WC, Ip P, Li GHY, Wong ICK, Kung AWC, Cheung CL: Maternal thyroid dysfunction during pregnancy and the risk of adverse outcomes in the offspring: A systematic review and meta-analysis. *Journal of Clinical Endocrinology and Metabolism* 2020, 105(12).

4. Li M: A meta-analysis of risk factors for congenital heart disease. *Chong Qing: Chinese Medical Association* 2010.

5. Lu RX: Risk factors for congenital heart disease in Chinese population: A meta-analysis. *Journal of Third military medical University* 2011, 33(12).

6. Hackshaw A, Rodeck C, Boniface S: Maternal smoking in pregnancy and birth defects: a systematic review based on 173 687 malformed cases and 11.7 million controls. *Human reproduction update* 2011, 17(5):589-604.

7. Nikfar S, Rahimi R, Hendoiee N, Abdollahi M: Increasing the risk of spontaneous abortion and major malformations in newborns following use of serotonin reuptake inhibitors during pregnancy: a systematic review and updated meta-analysis. *DARU Journal of Pharmaceutical Sciences* 2012, 20(1):1-10.

8. Wen J, Jiang J, Ding C, Dai J, Liu Y, Xia Y, Liu J, Hu Z: Birth defects in children conceived by in vitro fertilization and intracytoplasmic sperm injection: a meta-analysis. *Fertility and sterility* 2012, 97(6):1331-1337. e1334.

9. Dong JM: Risk factors and prenatal diagnostic methods for congenital heart disease: A meta-analysis. China: *Huazhong University of Science and Technology* 2013.

10. Xie SN: Relationship between Risk Factors during Pregnancy and Congenital Heart Disease in Children ：A Meta‐analysis. *Acta Medicinae Universitatis Scientiae et Technologiae Huazhong* 2013, 42(5).

11. Lee LJ, Lupo PJ: Maternal smoking during pregnancy and the risk of congenital heart defects in offspring: a systematic review and metaanalysis. *Pediatric cardiology* 2013, 34(2):398-407.

12. Painuly N, Painuly R, Heun R, Sharan P: Risk of cardiovascular malformations after exposure to paroxetine in pregnancy: meta-analysis. *The Psychiatrist* 2013, 37(6):198-203.

13. Riggin L, Frankel Z, Moretti M, Pupco A, Koren G: The fetal safety of fluoxetine: a systematic review and meta-analysis (vol 35, pg 362, 2013). *JOURNAL OF OBSTETRICS AND GYNAECOLOGY CANADA* 2013, 35(8):691-691.

14. Yakoob MY, Bateman BT, Ho E, Hernandez-Diaz S, Franklin JM, Goodman JE, Hoban RA: The risk of congenital malformations associated with exposure to β-blockers early in pregnancy: a meta-analysis. *Hypertension* 2013, 62(2):375-381.

15. Wu BS, Geng JS, Huang Y, Chen YY: Evaluation of protective effect of multivitamin on birth defects. *Chin Matern Child Health Care* 2014(14):2298-2300.

16. Chen EK-C, Zmirou-Navier D, Padilla C, Deguen S: Effects of air pollution on the risk of congenital anomalies: a systematic review and meta-analysis. *International journal of environmental research and public health* 2014, 11(8):7642-7668.

17. Nicoletti D, Appel LD, Siedersberger Neto P, Guimarães GW, Zhang L: Maternal smoking during pregnancy and birth defects in children: a systematic review with meta-analysis. *Cadernos de saude publica* 2014, 30:2491-2529.

18. Shi Q, Zhang J, Mi Y, Song Y, Ma J, Zhang Y: Congenital heart defects and maternal fever: systematic review and meta-analysis. *Journal of Perinatology* 2014, 34(9):677-682.

19. Alsaad AM, Kaplan YC, Koren G: Exposure to fluconazole and risk of congenital malformations in the offspring: a systematic review and meta-analysis. *Reproductive toxicology* 2015, 52:78-82.

20. De‐Regil LM, Peña‐Rosas JP, Fernández‐Gaxiola AC, Rayco‐Solon P: Effects and safety of periconceptional oral folate supplementation for preventing birth defects. *Cochrane database of systematic reviews* 2015(12).

21. Feng Y, Wang S, Chen R, Tong X, Wu Z, Mo X: Maternal folic acid supplementation and the risk of congenital heart defects in offspring: a meta-analysis of epidemiological observational studies. *Scientific reports* 2015, 5(1):1-8.

22. Simeone RM, Devine OJ, Marcinkevage JA, Gilboa SM, Razzaghi H, Bardenheier BH, Sharma AJ, Honein MA: Diabetes and congenital heart defects: a systematic review, meta-analysis, and modeling project. *American journal of preventive medicine* 2015, 48(2):195-204.

23. Sun J, Chen X, Chen H, Ma Z, Zhou J: Maternal alcohol consumption before and during pregnancy and the risks of congenital heart defects in offspring: a systematic review and meta‐analysis. *Congenital heart disease* 2015, 10(5):E216-E224.

24. Wang S, Yang L, Wang L, Gao L, Xu B, Xiong Y: Selective Serotonin Reuptake Inhibitors (SSRI s) and the Risk of Congenital Heart Defects: A Meta‐Analysis of Prospective Cohort Studies. *Journal of the American Heart Association* 2015, 4(5):e001681.

25. Yang J, Qiu H, Qu P, Zhang R, Zeng L, Yan H: Prenatal alcohol exposure and congenital heart defects: a meta-analysis. *PloS one* 2015, 10(6):e0130681.

26. Bérard A, Iessa N, Chaabane S, Muanda FT, Boukhris T, Zhao JP: The risk of major cardiac malformations associated with paroxetine use during the first trimester of pregnancy: a systematic review and meta‐analysis. *British journal of clinical pharmacology* 2016, 81(4):589-604.

27. Deguen S, Kihal W, Jeanjean M, Padilla C, Zmirou-Navier D: Neighborhood deprivation and risk of congenital heart defects, neural tube defects and orofacial clefts: A systematic review and meta-analysis. *PloS one* 2016, 11(10):e0159039.

28. Selmer R, Haglund B, Furu K, Andersen M, Nørgaard M, Zoëga H, Kieler H: Individual‐based versus aggregate meta‐analysis in multi‐database studies of pregnancy outcomes: the Nordic example of selective serotonin reuptake inhibitors and venlafaxine in pregnancy. *Pharmacoepidemiology and drug safety* 2016, 25(10):1160-1169.

29. Wen Z, Yu D, Zhang W, Fan C, Hu L, Feng Y, Yang L, Wu Z, Chen R, Yin K-j: Association between alcohol consumption during pregnancy and risks of congenital heart defects in offspring: meta-analysis of epidemiological observational studies. *Italian journal of pediatrics* 2016, 42(1):1-11.

30. Liu Y: Risk factors for congenital heart disease in Chinese neonates: a Meta analysis. *Chinese Journal of Contemporary Pediatrics* 2017, 19(7).

31. Gao SY, Wu QJ, Zhang TN, Shen ZQ, Liu CX, Xu X, Ji C, Zhao YH: Fluoxetine and congenital malformations: a systematic review and meta‐analysis of cohort studies. *British journal of clinical pharmacology* 2017, 83(10):2134-2147.

32. Zhang D, Cui H, Zhang L, Huang Y, Zhu J, Li X: Is maternal smoking during pregnancy associated with an increased risk of congenital heart defects among offspring? A systematic review and meta-analysis of observational studies. *The Journal of Maternal-Fetal & Neonatal Medicine* 2017, 30(6):645-657.

33. Li G: A meta-analysis of congenital heart disease and environmental risk factors in China from 2000 to 2016. *Anhui Medical Journal(China)* 2018, 39(4).

34. Xie GH: Association between Pre-pregnancy BMI and CongenitaI Heart Disease in Offspring: A Dose-response Meta-anaIysis. *Journal of Nursing**(China)* 2018, 25(15).

35. Munk-Olsen T, Liu X, Viktorin A, Brown HK, Di Florio A, D'Onofrio BM, Gomes T, Howard LM, Khalifeh H, Krohn H: Maternal and infant outcomes associated with lithium use in pregnancy: an international collaborative meta-analysis of six cohort studies. *The Lancet Psychiatry* 2018, 5(8):644-652.

36. Oldereid NB, Wennerholm U-B, Pinborg A, Loft A, Laivuori H, Petzold M, Romundstad LB, Söderström-Anttila V, Bergh C: The effect of paternal factors on perinatal and paediatric outcomes: a systematic review and meta-analysis. *Human reproduction update* 2018, 24(3):320-389.

37. Hall KC, Robinson JC: Association between maternal exposure to pollutant particulate matter 2.5 and congenital heart defects: a systematic review. *JBI database of systematic reviews and implementation reports* 2019, 17(8):1695.

38. Zhang Z, Zhang X, Zhou Yy, Jiang Cm, Jiang Hy: The safety of oral fluconazole during the first trimester of pregnancy: a systematic review and meta‐analysis. *BJOG: An International Journal of Obstetrics & Gynaecology* 2019, 126(13):1546-1552.

39. Li Q: Association between maternal alcohol consumption and risk of congenital heart disease in 0ffspring：a Meta analysis. *Chinese Journal of Contemporary Pediatrics* 2020, 22(6).

40. Fitton CA, Steiner MFC, Aucott L, Pell JP, Mackay DF, Fleming M, McLay JS: In utero exposure to antidepressant medication and neonatal and child outcomes: a systematic review. *Acta Psychiatr Scand* 2020, 141(1):21-33.

41. Joinau-Zoulovits F, Bertille N, Cohen JF, Khoshnood B: Association between advanced paternal age and congenital heart defects: a systematic review and meta-analysis. *Hum Reprod* 2020, 35(9):2113.

42. Picot C, Grenet G, Cucherat M, Cottin J: Pattern of malformations after methimazole or propylthiouracil exposure in early pregnancy: a meta-analytic approach. *Reproductive Toxicology* 2020, 97:6.

43. Papazoglou AS, Moysidis DV, Panagopoulos P, Kaklamanos EG, Tsagkaris C, Vouloagkas I, Karagiannidis E, Tagarakis GI, Papamitsou T, Papanikolaou IG *et al*: "Maternal diabetes mellitus and its impact on the risk of delivering a child with congenital heart disease: a systematic review and meta-analysis". *J Matern Fetal Neonatal Med* 2021:1-10.

44. Ravindra K, Chanana N, Mor S: Exposure to air pollutants and risk of congenital anomalies: A systematic review and metaanalysis. *Sci Total Environ* 2021, 765:142772.

45. Viswanathan M, Middleton JC, Stuebe A, Berkman N, Goulding AN, McLaurin-Jiang S, Dotson AB, Coker-Schwimmer M, Baker C, Voisin C *et al*: AHRQ Comparative Effectiveness Reviews. In: *Maternal, Fetal, and Child Outcomes of Mental Health Treatments in Women: A Systematic Review of Perinatal Pharmacologic Interventions.* edn. Rockville (MD): Agency for Healthcare Research and Quality (US); 2021.

**Supplementary Table S3 The characteristic of specific positive association between environment risk factors and CHD (n=185)**

|  | **ID** | **Study** | **Country** | **Factor** | **Outcome** | | **Number of studies included** | **Number of cases** | **OR or RR**  **(95% CI)** | **R/F** | **P** | **M** | **T** |
| --- | --- | --- | --- | --- | --- | --- | --- | --- | --- | --- | --- | --- | --- |
| **Reproductive related and assistive technologies** | | | |  | |  |  |  |  |  |  |  |  |
| 1 | 161 | Dong et al., 2013 | China | Family genetic history | | CHD | 5 observational studies | 7751 | 3.37 (2.68,4.22) | F | - | - | √ |
| 2 | 31 | Gijtenbeek et al., 2019 | Netherlands | MC twins vs. singletons | | CHD | 6 (3 prospective/3 retrospective) | NA | 6.32 (4.41,9.05) | F | - | √ | √ |
| 3 | 32 | Gijtenbeek et al., 2019 | Netherlands | MC twins with TTTS vs. singletons | | CHD | 6 (4 prospective/2 retrospective) | NA | 12.38(8.61,17.81) | R | - | √ | √ |
| 4 | 33 | Gijtenbeek et al., 2019 | Netherlands | MC twins without TTTS vs. singletons | | CHD | 5 (3 prospective/2 retrospective) | NA | 5.72 (3.81,8.59) | F | - | √ | √ |
| 5 | 34 | Gijtenbeek et al., 2019 | Netherlands | MC twins with TTTS vs. MC twins without TTTS | | CHD | 4 (2 prospective/2 retrospective) | NA | 2.39 (1.62,3.50) | F | - | √ | √ |
| 6 | 35 | Feng et al., 2014 | China | maternal parity (highest vs lowest) | | CHD | 14 (3 cohort/11 case-control) | NA | 2.10 (1.10,1.31) | R | √ | √ | √ |
| 7 | 37 | Feng et al., 2015 | China | Gravidity | | CHD | 10 (case-control) | NA | 1.18 (1.03,1.34) | R | √ | - | √ |
| 8 | 38 | Feng et al., 2016 | China | Gravidity number | | CHD | 8 (case-control) | NA | 1.13 (1.08,1.18) | F | √ | - | √ |
| 9 | 39 | Feng et al., 2017 | China | History of abortion | | CHD | 17 (case-control) | NA | 1.24 (1.11,1.38) | R | √ | - | √ |
| 10 | 40 | Feng et al., 2018 | China | History of spontaneous abortion | | CHD | 11 (case-control) | NA | 1.18 (1.07,1.31) | R | √ | - | √ |
| 11 | 41 | Feng et al., 2019 | China | History of induced abortion | | CHD | 6 (case-control) | NA | 1.24 (1.11,1.38) | R | √ | - | √ |
| 12 | 42 | Feng et al., 2020 | China | Abortion number | | CHD | 4 (case-control) | NA | 1.28 (1.18,1.40) | F | √ | - | √ |
| 13 | 43 | Giorgione et al., 2018 | Italy | ICSI/IVF pregnancies | | CHD | 8 (cohort) | 287995 | 1.45 (1.20,1.76) | R | √ | √ | √ |
| 14 | 47 | Giorgione et al., 2018 | Italy | Singleton IVF/ICSI | | CHD | 5 (cohort) | 13396 | 1.55 (1.21,1.99) | R | √ | √ | √ |
| 15 | 49 | Ou et al., 2018 | China | ICSI vs IVF (in fresh transplantation cycle) | | CHD | 3 (cohort) | 72 | 2.07 (1.28,3.37) | F | √ | √ | √ |
| 16 | 54 | Ma et al., 2014 | China | History of induced abortion | | CHD | 3 (case-control) | 476 | 2.88 (1.88,4.40) | F | √ | √ | √ |
| 17 | 55 | Ma et al., 2015 | China | Maternal or fetal abnormalities detected | | CHD | 3 (case-control) | 364 | 2.31 (1.25,4.27) | F | √ | √ | √ |
| **Parental age and BMI** | | |  |  | |  |  |  |  |  |  |  |  |
| 18 | 64 | Wang et al., 2015 | China | Advanced maternal age (≥35 years) | | CHD | 9 (case-control) | 19201 | 1.13 (1.07,1.18) | F | √ | √ | √ |
| 19 | 69 | Peng et al., 2019 | China | Paternal age (35–39 years) | | CHD | 5 (case-control) | 2816 | 1.14 (1.09,1.19) | R | √ | √ | √ |
| 20 | 70 | Fang et al., 2020 | China | Paternal age (≥40 years) | | CHD | 11 observational studies | 32190 | 1.10 (1.01,1.20) | R | √ | √ | √ |
| 21 | 73 | Zheng et al., 2018 | China | Maternal overweight | | CHD | 19 (3 cohort/16 case-control) | NA | 1.07 (1.00,1.13) | R | √ | √ | √ |
| 22 | 74 | Zheng et al., 2018 | China | Maternal obesity | | CHD | 20 (5 cohort/15 case-control) | NA | 1.32 (1.21,1.43) | R | √ | √ | √ |
| 23 | 75 | Stothard et al., 2009 | UK | Maternal obesity | | Septal defects | 4 (case-control) | 3483 | 1.20 (1.09,1.31) | F | √ | √ | √ |
| 24 | 78 | Cai et al., 2014 | China | Maternal moderate obesity | | CHD | 5 observational studies | 18146 | 1.15 (1.09,1.21) | F | √ | - | √ |
| 25 | 79 | Cai et al., 2014 | China | Maternal severe obesity | | CHD | 5 observational studies | 16020 | 1.38 (1.30,1.47) | F | √ | - | √ |
| 26 | 81 | Cai et al., 2014 | China | Overweight | | HLHS | 4 observational studies | 490 | 1.31 (1.08,1.59) | F | √ | - | √ |
| 27 | 82 | Cai et al., 2014 | China | Moderate obesity | | HLHS | 3 observational studies | 421 | 1.54 (1.21,1.94) | F | √ | - | √ |
| 28 | 83 | Cai et al., 2014 | China | Severe obesity | | HLHS | 3 observational studies | 361 | 1.56 (1.08,2.27) | F | √ | - | √ |
| 29 | 84 | Cai et al., 2014 | China | Obesity | | HLHS | 4 observational studies | 467 | 1.51 (1.23,1.86) | F | √ | - | √ |
| 30 | 86 | Cai et al., 2014 | China | Overweight | | OFT | 3 observational studies | 2344 | 1.19 (1.09,1.31) | F | √ | - | √ |
| 31 | 87 | Cai et al., 2014 | China | Obesity | | OFT | 3 observational studies | 2206 | 1.39 (1.25,1.55) | F | √ | - | √ |
| 32 | 90 | Cai et al., 2014 | China | Moderate obesity | | ASD | 3 observational studies | 2167 | 1.26 (1.14,1.41) | F | √ | - | √ |
| 33 | 91 | Cai et al., 2014 | China | Severe obesity | | ASD | 3 observational studies | 1880 | 1.67 (1.40,1.99) | F | √ | - | √ |
| 34 | 92 | Cai et al., 2014 | China | Obesity | | ASD | 4 observational studies | 2328 | 1.35 (1.23,1.49) | F | √ | - | √ |
| 35 | 96 | Cai et al., 2014 | China | Severe obesity | | TOF | 3 observational studies | 648 | 1.94 (1.49,2.51) | F | √ | - | √ |
| 36 | 97 | Cai et al., 2014 | China | Obesity | | TOF | 5 observational studies | 887 | 1.28 (1.09,1.50) | F | √ | - | √ |
| 37 | 100 | Cai et al., 2014 | China | Obesity | | CTD | 3 observational studies | 1278 | 1.22 (1.07,1.40) | F | √ | - | √ |
| 38 | 104 | Cai et al., 2014 | China | Severe obesity | | AVSD | 3 observational studies | 756 | 1.43 (1.03,2.00) | F | √ | - | √ |
| 39 | 109 | Cai et al., 2014 | China | Severe obesity | | VSD | 3 observational studies | 4409 | 1.23 (1.07,1.41) | F | √ | - | √ |
| 40 | 113 | Cai et al., 2014 | China | Moderate obesity | | COA | 3 observational studies | 502 | 1.29 (1.03,1.61) | F | √ | - | √ |
| 41 | 115 | Cai et al., 2014 | China | Obesity | | COA | 4 observational studies | 534 | 1.24 (1.02,1.53) | F | √ | - | √ |
| **Parental life habits, working and dwelling environment** | | | | | |  |  |  |  |  |  |  |  |
| 42 | 121 | Zhao et al., 2020 | China | Maternal active smoking | | CHD | 86 observational studies | 137574 | 1.25 (1.16,1.34) | B | √ | √ | √ |
| 43 | 123 | Zhao et al., 2020 | China | Maternal active smoking | | ASD | 9 observational studies | NA | 1.27 (1.02,1.59) | R | √ | √ | √ |
| 44 | 127 | Zhao et al., 2020 | China | Maternal active smoking | | RVOTO | 4 observational studies | NA | 1.43 (1.04,1.97) | F | √ | √ | √ |
| 45 | 132 | Zhao et al., 2020 | China | Maternal passive smoking | | CHD | 36 observational studies | NA | 2.24 (1.81,2.77) | R | √ | √ | √ |
| 46 | 133 | Zhao et al., 2020 | China | Paternal active smoking | | CHD | 35 observational studies | NA | 1.93 (1.24,3.00) | R | √ | √ | √ |
| 47 | 134 | Peng et al., 2019 | China | Paternal smoking (cigarette/day) | | CHD | 10 (case-control) | 8709 | 1.42 (1.17,1.74) | R | √ | √ | √ |
| 48 | 136 | Peng et al., 2019 | China | Paternal Smoking (10–19 cigarette/day) | | CHD | 4 (case-control) | 467 | 1.41 (1.20,1.67) | R | √ | √ | √ |
| 49 | 137 | Peng et al., 2019 | China | Paternal Smoking (≥20 cigarette/day) | | CHD | 5 (case-control) | 1131 | 1.75 (1.10,2.80) | R | √ | √ | √ |
| 50 | 138 | Zhang et al., 2019 | China | Maternal alcohol consumption | | CHD | 45 observational studies | NA | 1.16 (1.05,1.27) | R | √ | √ | - |
| 51 | 143 | Zhang et al., 2019 | China | Maternal alcohol consumption | | TOF | 6 observational studies | NA | 1.20 (1.08,1.33) | F | √ | √ | - |
| 52 | 147 | Zhang et al., 2019 | China | Paternal alcohol consumption | | CHD | 24 observational studies | NA | 1.44 (1.19,1.74) | R | √ | √ | - |
| 53 | 57 | Yue et al., 2021 | China | Caffeinated products | | CHD | 10 (1 cohort/8 case-control/1 cross-section) | 9700 | 1.17 (1.07,1.28) | F | √ | √ | - |
| 54 | 60 | Yue et al., 2021 | China | High intake of caffeinated products | | CHD | 4 (1 cohort/3 case-control) | 7800 | 1.32 (1.09,1.60) | F | √ | √ | √ |
| 55 | 148 | Spinder et al., 2019 | Netherlands | Solvents exposure | | CHD | 6 (case-control) | 2526 | 1.31 (1.06,1.63) | F | √ | √ | √ |
| 56 | 153 | Yu et al., 2014 | China | Maternal educational attainment | | CHD | 29 (2 cohort/27 case-control) | NA | 1.11 (1.03,1.21) | R | √ | √ | √ |
| 57 | 154 | Yu et al., 2014 | China | Family income | | CHD | 5 (1 cohort/4 case-control) | NA | 1.05 (1.01,1.09) | R | √ | √ | √ |
| 58 | 155 | Dong et al., 2013 | China | Paternal occupational exposure to adverse substances | | CHD | 3 observational studies | 919 | 1.65 (1.18,2.29) | F | - | - | √ |
| 59 | 157 | Zhou et al., 2016 | China | Maternal occupational risk factors | | CHD | 5(case-control) | 8588 | 5.14 (3.30,8.00) | F | √ | √ | - |
| 60 | 159 | Zhou et al., 2016 | China | Decoration materials during pregnancy | | CHD | 3(case-control) | 8588 | 4.14 (2.27,7.58) | R | √ | √ | √ |
| 61 | 262 | Fornaro et al.,2020 | Italy | Lithium exposure (first trimester vs. general population) | | CHD | 4 (cohort) | 1436 | 1.96 (1.28,3.00) | R | √ | √ | √ |
| 62 | 263 | Fornaro et al.,2020 | Italy | Lithium exposure (first trimester vs. unexposed women) | | CHD | 3 (cohort) | 815 | 3.99 (1.19,13.43) | R | √ | √ | √ |
| 63 | 264 | Fornaro et al.,2020 | Italy | Lithium exposure (first trimester vs. patients with bipolar disordered) | | CHD | 4 (cohort) | 1436 | 1.75 (1.08,2.84) | R | √ | √ | √ |
| 64 | 374 | Jiang et al.,2019 | China | Exposure to noise during pregnancy | | CHD | 5 (case-control) | NA | 3.19 (2.37,4.29) | R | √ | √ | √ |
| 65 | 375 | Zhou et al.,2016 | China | Malnutrition during pregnancy | | CHD | 4 (case-control) | 8588 | 1.75 (1.39,2.22) | F | √ | √ | √ |
| **Maternal drug exposure** | | |  |  | |  |  |  |  |  |  |  |  |
| 66 | 163 | Xu et al., 2016 | China | Folic acid supplementation | | CHD | 20 (case-control) | NA | 0.60 (0.49,0.71) | B | √ | √ | √ |
| 67 | 162 | Tanoshima et al., 2015 | Japan | Valproic acid in utero | | CHD | 59 (cohort) | NA | 2.08 (1.55,2.79) | B | √ | √ | - |
| 68 | 164 | Budani et al., 2021 | Italy | Fluconazole (in the first trimester) | | CHD | 5 (RCT)* | 24680 | 1.95 (1.18,3.21) | R | - | - | √ |
| 69 | 166 | Zhang et al., 2019 | China | Fluconazole (in the first trimester) | | Septal defects | 3 (1 cohort/2 case-control) | NA | 1.30 (1.00,1.67) | F | √ | √ | √ |
| 70 | 170 | Picot et al., 2020 | France | Ondansetro | | HLHS | 3 observational studies | NA | 1.49 (1.03,2.17) | F | √ | √ | √ |
| 71 | 173 | Carl et al., 2018 | UK | Oral hormone pregnancy tests | | CHD | 7(2 cohort/5 case-control) | 62 | 1.89 (1.32,2.27) | F | √ | √ | √ |
| 72 | 177 | Nieuwenhuijsen et al., 2009 | Spain | High vs. low chlorination by-products | | VSD | 3 observational studies | NA | 1.59 (1.21,2.07) | F | √ | - | - |
| 73 | 179 | Goldberg et al., 2015 | Canada | Nitrofurantoin during early pregnancy | | HLHS | 3 (1 case-control/2 cohort) | 305 | 3.07 (1.59,5.93) | R | √ | - | √ |
| 74 | 182 | Zhang et al., 2017 | China | SSRIs (in the first trimester) | | Cardiovascular related malformations | 18 (cohort) | NA | 1.26 (1.13,1.39) | R | √ | √ | √ |
| 75 | 184 | Zhang et al., 2017 | China | SSRIs (in the first trimester) | | ASD | 6 (cohort) | NA | 2.06 (1.40,3.03) | R | √ | √ | √ |
| 76 | 186 | Zhang et al., 2017 | China | SSRIs (in the first trimester) | | ASD and/or VSD | 14 (cohort) | NA | 1.27 (1.14,1.42) | R | √ | √ | √ |
| 77 | 187 | Gao et al., 2018 | China | SSRIs | | Septal defects | 6 (cohort) | 20201 | 1.38 (1.00,1.91) | R | √ | √ | √ |
| 78 | 188 | Gao et al., 2018 | China | SSRIs | | ASD | 7 (cohort) | 35554 | 1.83 (1.22,2.73) | R | √ | √ | √ |
| 79 | 190 | Gao et al., 2018 | China | SSRIs | | RVOTO | 4 (cohort) | 97575 | 1.38 (1.09,1.75) | R | √ | √ | √ |
| 80 | 193 | Gao et al., 2018 | China | Citalopram | | CHD | 11 (cohort) | 26374 | 1.24 (1.02,1.51) | R | √ | √ | √ |
| 81 | 194 | Gao et al., 2018 | China | Citalopram | | Septal defects | 3 (cohort) | 2724 | 1.81 (1.22,2.68) | R | √ | √ | - |
| 82 | 200 | Gao et al., 2018 | China | Fluoxetine | | CHD | 14 (cohort) | 28959 | 1.30 (1.12,1.53) | R | √ | √ | √ |
| 83 | 201 | Gao et al., 2018 | China | Fluoxetine | | Septal defects | 3 (cohort) | 1748 | 1.65 (1.02,2.67) | R | √ | √ | - |
| 84 | 204 | Gao et al., 2018 | China | Fluoxetine | | RVOTO | 2 (cohort) | 12866 | 1.63 (1.11,2.41) | R | √ | √ | - |
| 85 | 207 | Gao et al., 2018 | China | Paroxetine | | CHD | 16 (cohort) | 22770 | 1.35 (1.19,1.53) | R | √ | √ | √ |
| 86 | 211 | Gao et al., 2018 | China | Paroxetine | | RVOTO | 2 (cohort) | 12094 | 2.15 (1.04,4.44) | R | √ | √ | - |
| 87 | 214 | Gao et al., 2018 | China | Sertraline | | CHD | 13 (cohort) | 29309 | 1.42 (1.12,1.80) | R | √ | √ | √ |
| 88 | 215 | Gao et al., 2018 | China | Sertraline | | Septal defects | 4 (cohort) | 1428 | 2.69 (1.76,4.10) | R | √ | √ | √ |
| 89 | 216 | Gao et al., 2018 | China | Sertraline | | ASD | 2 (cohort) | 1686 | 2.07 (1.26,3.39) | R | √ | √ | - |
| 90 | 230 | De Vries et al.,2021 | Australia | Any antidepressant (in the first trimester) | | CHD | 20 (15 cohort/5 case-control) | 61539 | 1.28 (1.17,1.41) | R | √ | √ | √ |
| 91 | 231 | De Vries et al.,2021 | Australia | SSRIs | | CHD | 16 (14 cohort/2 case-control) | 43170 | 1.25 (1,15,1.37) | R | √ | √ | √ |
| 92 | 232 | De Vries et al.,2021 | Australia | SNRI | | CHD | 4 (3 cohort/1 case-control) | 24743 | 1.69 (1.37,2.10) | R | √ | √ | √ |
| 93 | 235 | De Vries et al.,2021 | Australia | Bupropion | | CHD | 3 (1 cohort/2 case-control) | 6591 | 1.23 (1.01,1.49) | R | √ | √ | √ |
| 94 | 236 | Wu et al.,2021 | China | ß−blockers (in the first trimester) | | CHD | 9 (5 cohort/ 4case-control) | NA | 1.29 (1.02,1.62) | R | √ | √ | √ |
| 95 | 238 | Picot et al.,2020 | France | Methimazole | | CHD | 3 observational studies | NA | 1.73 (1.40,2.15) | R | - | - | - |
| 96 | 239 | Huo et al.,2013 | China | Antibiotics | | CHD | 5 (case-control) | 853 | 2.49 (1.80,3.45) | F | √ | √ | - |
| 97 | 244 | Rahimi et al.,2021 | Iran | Nitrate (high vs low) | | CHD | 4 (case-control) | 136 | 1.20 (1.01,1.42) | R | √ | √ | √ |
| 98 | 245 | Rahimi et al.,2021 | Iran | Nitrate (each additional daily 0.5 mg) | | CHD | 3 (case-control) | 131 | 1.03 (1.00,1.05) | F | √ | √ | √ |
| **Mantal disease** | | |  |  | |  |  |  |  |  |  |  |  |
| 99 | 246 | Ye et al.,2019 | China | Viral infection | | CHD | 17 (case-control) | 67233 | 1.83 (1.58,2.12) | B | √ | √ | √ |
| 100 | 249 | Ye et al.,2019 | China | Cytomegalovirus infection | | CHD | 4 (case-control) | NA | 3.95 (1.87,8.36) | B | √ | √ | √ |
| 101 | 253 | Ye et al.,2019 | China | Rubella virus | | CHD | 7 (case-control) | NA | 3.54 (1.75,7.15) | B | √ | √ | √ |
| 102 | 265 | Yang et al.,2021 | China | Fever | | CHD | 16 (1 cohort/15 case-control) | 31922 | 1.45 (1.21,1.73) | R | √ | √ | √ |
| 103 | 266 | Yang et al.,2021 | China | Fever | | CTD | 4 (4case-control) | 13154 | 1.38 (1.01,1.89) | R | √ | √ | √ |
| 104 | 267 | Yang et al.,2021 | China | Fever | | ASD | 6 (case-control) | 18479 | 1.48 (1.01,2.17) | R | √ | √ | √ |
| 105 | 269 | Yang et al.,2021 | China | Fever | | TGA | 4 (case-control) | 17150 | 1.81 (1.14,2.88) | R | √ | √ | √ |
| 106 | 273 | Yang et al.,2021 | China | Fever | | RVOTO | 3 (case-control) | 13071 | 1.66 (1.04,2.65) | R | √ | √ | √ |
| 107 | 274 | Luteijn et al.,2014 | UK | Influenza | | CHD | 10 observational studies | 7715 | 1.56 (1.13,2.14) | R | √ | √ | √ |
| 108 | 275 | Luteijn et al.,2014 | UK | Influenza | | AVA/AVS | 3 observational studies | 167 | 2.03 (1.27,3.27) | R | √ | √ | - |
| 109 | 276 | Luteijn et al.,2014 | UK | Influenza | | VSD | 3 observational studies | 429 | 1.59 (1.24,2.14) | R | √ | √ | - |
| 110 | 277 | Hoang et al.,2017 | USA | DM | | CHD | 12 observational studies | 72828 | 3.59 (3.03,4.50) | R | - | - | - |
| 111 | 278 | Hoang et al.,2017 | USA | DM | | ASD | NA | 2515 | 2.75 (1.66,4.54) | R | - | - | - |
| 112 | 279 | Hoang et al.,2017 | USA | DM | | AVCD | NA | 642 | 5.78 (3.69,9.06) | F | - | - | - |
| 113 | 281 | Hoang et al.,2017 | USA | DM | | TOF | NA | 2441 | 4.14 (2.09,8.20) | R | - | - | - |
| 114 | 282 | Hoang et al.,2017 | USA | DM | | LVOTO | NA | 1672 | 14.49(8.29,25.34) | F | - | - | - |
| 115 | 283 | Hoang et al.,2017 | USA | DM | | Aortic stenosis | NA | 209 | 3.51 (2.63,4.70) | F | - | - | - |
| 116 | 284 | Hoang et al.,2017 | USA | DM | | COA | NA | 823 | 3.38 (2.34,4.87) | F | - | - | - |
| 117 | 285 | Hoang et al.,2017 | USA | DM | | HLHS | NA | 460 | 3.26 (1.98,5.39) | F | - | - | - |
| 118 | 286 | Hoang et al.,2017 | USA | DM | | RVOTO | NA | 1387 | 3.33 (2.55,4.34) | F | - | - | - |
| 119 | 287 | Hoang et al.,2017 | USA | DM | | PVA | NA | 72 | 3.91 (2.16,7.07) | F | - | - | - |
| 120 | 288 | Hoang et al.,2017 | USA | DM | | PVS | NA | 2346 | 2.75 (2.00,3.76) | F | - | - | - |
| 121 | 289 | Hoang et al.,2017 | USA | DM | | TAVPR | NA | 141 | 3.66 (1.90,7.06) | F | - | - | - |
| 122 | 290 | Hoang et al.,2017 | USA | DM | | VSD | NA | 5037 | 2.76 (2.20,3.47) | R | - | - | - |
| 123 | 291 | Chen et al.,2019 | China | DM | | CHD | 52 (16 cohort/36 case-control) | 259917 | 2.71 (2.28,3.23) | R | √ | √ | √ |
| 124 | 292 | Chen et al.,2019 | China | PGDM | | CHD | 31 observational studies | NA | 3.18 (2.77,3.65) | R | √ | √ | √ |
| 125 | 293 | Chen et al.,2019 | China | GDM | | CHD | 27 observational studies | NA | 1.98 (1.66,2.36) | R | √ | √ | √ |
| 126 | 294 | Chen et al.,2019 | China | DM | | heterotaxia | 6 observational studies | NA | 4.29 (2.09,8.78) | R | √ | √ | - |
| 127 | 295 | Chen et al.,2019 | China | DM | | AVCD | 3 observational studies | NA | 2.48 (2.06,2.99) | R | √ | √ | - |
| 128 | 296 | Chen et al.,2019 | China | DM | | PDA | 3 observational studies | NA | 4.22 (2.79,6.40) | R | √ | √ | - |
| 129 | 297 | Chen et al.,2019 | China | DM | | CTD | 6 observational studies | NA | 3.81 (2.62,5.55) | R | √ | √ | - |
| 130 | 298 | Chen et al.,2019 | China | DM | | TA | 5 observational studies | NA | 5.06 (2.65,9.65) | R | √ | √ | - |
| 131 | 299 | Chen et al.,2019 | China | DM | | TGA | 12 observational studies | NA | 2.58 (1.99,3.36) | R | √ | √ | - |
| 132 | 300 | Chen et al.,2019 | China | DM | | TOF | 9 observational studies | NA | 2.88 (1.87,4.44) | R | √ | √ | - |
| 133 | 302 | Chen et al.,2019 | China | DM | | AVSD | 5 observational studies | NA | 5.74 (3.20,10.27) | R | √ | √ | - |
| 134 | 303 | Chen et al.,2019 | China | DM | | APVR | 5 observational studies | NA | 2.75 (1.30,5.85) | R | √ | √ | - |
| 135 | 304 | Chen et al.,2019 | China | DM | | LVOTO | 10 observational studies | NA | 2.78 (1.92,4.02) | R | √ | √ | - |
| 136 | 305 | Chen et al.,2019 | China | DM | | COA | 9 observational studies | NA | 2.33 (2.00,2.70) | R | √ | √ | - |
| 137 | 306 | Chen et al.,2019 | China | DM | | VAS | 7 observational studies | NA | 2.46 (1.96,4.02) | R | √ | √ | - |
| 138 | 307 | Chen et al.,2019 | China | DM | | HLHS | 8 observational studies | NA | 1.80 (1.40,2.03) | R | √ | √ | - |
| 139 | 308 | Chen et al.,2019 | China | DM | | RVOTO | 8 observational studies | NA | 2.78 (1.77,4.38) | R | √ | √ | - |
| 140 | 310 | Chen et al.,2019 | China | DM | | PAS | 8 observational studies | NA | 2.59 (1.93,3.47) | R | √ | √ | - |
| 141 | 313 | Chen et al.,2019 | China | DM | | Septal defects | 3 observational studies | NA | 3.08 (2.23,4.24) | R | √ | √ | - |
| 142 | 314 | Chen et al.,2019 | China | DM | | VSD | 16 observational studies | NA | 2.43 (1.90,3.11) | R | √ | √ | - |
| 143 | 315 | Chen et al.,2019 | China | DM | | ASD | 14 observational studies | NA | 2.70 (1.99,3.68) | R | √ | √ | - |
| 144 | 316 | Chen et al.,2019 | China | DM | | VSD+ASD | 6 observational studies | NA | 2.97 (2.01,4.39) | R | √ | √ | - |
| 145 | 317 | Chen et al.,2019 | China | DM | | Valve defects | 5 observational studies | NA | 2.51 (2.16,2.92) | R | √ | √ | - |
| 146 | 318 | Chen et al.,2019 | China | DM | | Single ventricle | 3 observational studies | NA | 3.46 (2.63,4.56) | R | √ | √ | - |
| 147 | 319 | Chen et al.,2019 | China | PGDM | | heterotaxia | 5 observational studies | NA | 7.80 (5.64,10.78) | R | √ | √ | - |
| 148 | 320 | Chen et al.,2019 | China | PGDM | | AVCD | 3 observational studies | NA | 6.12 (4.05,9.26) | R | √ | √ | - |
| 149 | 321 | Chen et al.,2019 | China | PGDM | | PDA | 3 observational studies | NA | 4.22 (2.79,6.40) | R | √ | √ | - |
| 150 | 322 | Chen et al.,2019 | China | PGDM | | CTD | 6 observational studies | NA | 3.81 (2.62,5.55) | R | √ | √ | - |
| 151 | 323 | Chen et al.,2019 | China | PGDM | | TA | 5 observational studies | NA | 7.81 (3.50,17.43) | R | √ | √ | - |
| 152 | 324 | Chen et al.,2019 | China | PGDM | | TGA | 9 observational studies | NA | 3.67 (2.63,5.12) | R | √ | √ | - |
| 153 | 325 | Chen et al.,2019 | China | PGDM | | TOF | 7 observational studies | NA | 4.25 (3.02,10.27) | R | √ | √ | - |
| 154 | 327 | Chen et al.,2019 | China | PGDM | | AVSD | 5 observational studies | NA | 5.74 (3.20,10.27) | R | √ | √ | - |
| 155 | 328 | Chen et al.,2019 | China | PGDM | | APVR | 4 observational studies | NA | 3.94 (2.45,6.34) | R | √ | √ | - |
| 156 | 329 | Chen et al.,2019 | China | PGDM | | LVOTO | 8 observational studies | NA | 3.93 (3.11,4.98) | R | √ | √ | - |
| 157 | 330 | Chen et al.,2019 | China | PGDM | | COA | 7 observational studies | NA | 3.66 (2.60,5.16) | R | √ | √ | - |
| 158 | 331 | Chen et al.,2019 | China | PGDM | | VAS | 5 observational studies | NA | 4.04 (2.60,6.30) | R | √ | √ | - |
| 159 | 332 | Chen et al.,2019 | China | PGDM | | HLHS | 5 observational studies | NA | 3.49 (1.90,6.42) | R | √ | √ | - |
| 160 | 333 | Chen et al.,2019 | China | PGDM | | RVOTO | 7 observational studies | NA | 4.01 (3.65,4.39) | R | √ | √ | - |
| 161 | 335 | Chen et al.,2019 | China | PGDM | | PAS | 7 observational studies | NA | 3.75 (2.88,4.89) | R | √ | √ | - |
| 162 | 338 | Chen et al.,2019 | China | PGDM | | Septal defects | 3 observational studies | NA | 3.63 (3.28,4.02) | R | √ | √ | - |
| 163 | 339 | Chen et al.,2019 | China | PGDM | | VSD | 13 observational studies | NA | 3.07 (2.52,3.75) | R | √ | √ | - |
| 164 | 340 | Chen et al.,2019 | China | PGDM | | ASD | 11 observational studies | NA | 3.23 (2.49,4.19) | R | √ | √ | - |
| 165 | 341 | Chen et al.,2019 | China | PGDM | | VSD+ASD | 5 observational studies | NA | 3.31 (2.85,3.85) | R | √ | √ | - |
| 166 | 342 | Chen et al.,2019 | China | PGDM | | Valve defects | 5 observational studies | NA | 3.96 (3.25,4.83) | R | √ | √ | - |
| 167 | 343 | Chen et al.,2019 | China | PGDM | | Single ventricle | 3 observational studies | NA | 4.28 (2.08,8.83) | R | √ | √ | - |
| 168 | 349 | Chen et al.,2019 | China | GDM | | TOF | 4 observational studies | NA | 1.50 (1.11,2.03) | R | √ | √ | √ |
| 169 | 352 | Chen et al.,2019 | China | GDM | | LVOTO | 3 observational studies | NA | 1.81 (1.50,2.18) | R | √ | √ | - |
| 170 | 353 | Chen et al.,2019 | China | GDM | | COA | 4 observational studies | NA | 2.08 (1.70,2.54) | R | √ | √ | - |
| 171 | 354 | Chen et al.,2019 | China | GDM | | VAS | 5 observational studies | NA | 1.78 (1.26,2.51) | R | √ | √ | - |
| 172 | 358 | Chen et al.,2019 | China | GDM | | PAS | 4 observational studies | NA | 1.89 (1.17,3.05) | R | √ | √ | - |
| 173 | 362 | Chen et al.,2019 | China | GDM | | VSD | 5 observational studies | NA | 1.34 (1.21,1.47) | R | √ | √ | - |
| 174 | 363 | Chen et al.,2019 | China | GDM | | ASD | 5 observational studies | NA | 1.86 (1.29,2.69) | R | √ | √ | - |
| 175 | 364 | Chen et al.,2019 | China | GDM | | VSD+ASD | 3 observational studies | NA | 1.37 (1.24,1.50) | R | √ | √ | - |
| 176 | 365 | Chen et al.,2019 | China | GDM | | Valve defects | 3 observational studies | NA | 1.87 (1.50,2.34) | R | √ | √ | - |
| 177 | 366 | Ma et al.,2017 | China | Gestational hypertension | | CHD | 23 (10 cohort/13 case-control) | NA | 1.56 (1.50,1.83) | R | √ | √ | √ |
| 178 | 369 | Ma et al.,2017 | China | Gestational hypertension | | VSD | 4 observational studies | NA | 2.05(1.12,3.73) | R | √ | √ | √ |
| 179 | 370 | Ma et al.,2017 | China | Gestational hypertension | | ASD | 6 observational studies | NA | 2.25 (1.35,3.74) | R | √ | √ | - |
| 180 | 371 | Ma et al.,2017 | China | Gestational hypertension | | AVS | 5 observational studies | NA | 1.48 (1.16,1.89) | F | √ | √ | - |
| 181 | 372 | Zhu et al.,2017 | China | Mental stimulation | | CHD | 11 (case-control) | NA | 2.93 (1.72,4.98) | R | √ | - | - |
| 182 | 373 | Jiang et al.,2019 | China | Exposure to harmful chemicals during pregnancy | | CHD | 13 (case-control) | NA | 3.59 (2.41,5.36) | R | √ | √ | √ |
| 183 | 376 | Zhou et al.,2016 | China | Respiratory infection during pregnancy | | CHD | 5 (case-control) | 8588 | 3.78 (1.93,7.38) | R | √ | √ | √ |
| 184 | 377 | Zhou et al.,2016 | China | Infection of the reproductive system | | CHD | 3 (case-control) | 8588 | 4.68 (1.06,20.53) | R | √ | √ | √ |
| 185 | 378 | Zhou et al.,2016 | China | Chronic diseases before pregnancy | | CHD | 3 (case-control) | 8588 | 4.41 (2.14,9.09) | F | √ | √ | √ |

**Abbreviation**: OR, Odds ratio; RR, relative risk; CI, Confidence interval; R:Random-effects model; F:Fixed-effects model; P:Publication bias assessment; M:Methodological evaluation; T:Traceability;NA: Not available; IVF: In-vitro-fertilization; ICSI: Intracytoplasmic sperm injection; TTHM, Total trihalomethane; SSRI, Selective serotonin reuptake inhibitor; TCA, Tricyclic antidepressant; NSAIDs, Nonsteroidal anti-inflammatory drugs; HPTs, hormone pregnancy tests; DM, maternal diabetes mellitus; PGDM, pregestational diabetes mellitus; GDM, gestational diabetes mellitus; TTTS, twin–twin transfusion syndrome; MC, Monochorionic; SNRI, serotonin-norepinephrine reuptake inhibitor; ASD, Atrial septal defect; TOF, Tetralogy of fallot; VSD, Ventricular septal defects; CTD, Conotruncal defects; COA, Coarctation of the aorta; PDA, Patent ductus arteriosus; PVS, Pulmonary valve stenosis; CHD, Congenital heart disease; TOF, Tetralogy of fallot; TGA, Transposition of great arteries; HLHS, Hypoplastic left heart syndrome; OFT, outflow tract; AVSD, Atrioventricular septal defect; LVOTO, Left ventricular outflow tract obstruction; RVOTO, Right ventricular outflow tract obstruction; PVA, Pulmonary valve atresia; TAPVR, Total anomalous pulmonary venous return; TA, Truncus arteriosus; APVR, Anomalous pulmonary venous return; VAS, Valvular aortic stenosis; PAS, Pulmonary artery stenosis; AVA/AVS, Aortic valve atresia/stenosis.

**Supplementary Table S4 The characteristic of specific negative association between environment risk factors and CHD (n=136)**

| **ID** | **Author** | **Country** | **Year of publication** | | **Exposure** | **Outcome** | **Number of studies included** | **Number of cases** | | **OR/RR (95% CI)** |
| --- | --- | --- | --- | --- | --- | --- | --- | --- | --- | --- |
| **Air pollution** | | | | | | | | | | |
| 1 | Hu et al.,2020 | China、Netherlands | 2020 | CO per 1 ppm（Continuous exposure） | | ASD | 9(2 cohort/7 case-control) | 2220 | | 0.85 (0.72, 1.01) |
| 2 | Hu et al.,2020 | China、Netherlands | 2020 | CO per 1 ppm（Continuous exposure） | | TOF | 8(2 cohort/6 case-control) | 688 | | 1.57 (0.93, 2.64) |
| 3 | Hu et al.,2020 | China、Netherlands | 2020 | CO per 1 ppm（Continuous exposure） | | VSD | 11(3 cohort/8 case-control) | 4652 | | 1.04 (0.69, 1.56) |
| 4 | Li et al.,2017 | China | 2017 | CO | | CTD | 5 (1 cohort/4 case-control) | NA* | | 0.77 (0.44, 1.33) |
| 5 | Hu et al.,2020 | China、Netherlands | 2020 | NO_2_ per 10 ppb（Continuous exposure） | | ASD | 12(4 cohort/8 case-control） | 2871 | | 0.98 (0.90, 1.06) |
| 6 | Hu et al.,2020 | China、Netherlands | 2020 | NO_2_ per 10 ppb（Continuous exposure） | | COA | 9(4cohort/5 case-control） | 927 | | 1.12 (0.99, 1.28) |
| 7 | Hu et al.,2020 | China、Netherlands | 2020 | NO_2_ per 10 ppb（Continuous exposure） | | PDA | 4(3cohort/1 case-control） | 625 | | 1.10 (0.92, 1.31) |
| 8 | Hu et al.,2020 | China、Netherlands | 2020 | NO_2_ per 10 ppb（Continuous exposure） | | PVS | 6(3cohort/3 case-control） | 679 | | 0.91 (0.72, 1.15) |
| 9 | Hu et al.,2020 | China、Netherlands | 2020 | NO_2_ per 10 ppb（Continuous exposure） | | TOF | 11(5cohort/6 case-control） | 969 | | 1.11 (0.95, 1.30) |
| 10 | Hu et al.,2020 | China、Netherlands | 2020 | NO_2_ per 10 ppb（Continuous exposure） | | VSD | 13(5 cohort/8 case-control） | 5397 | | 0.97 (0.91, 1.04) |
| 11 | Zhang et al., 2018 | China | 2018 | NO_X_ per 10 ppb Increment | | ASD | 8 (2cohort/6case-control) | NA | | 1.01 (0.96, 1.06) |
| 12 | Zhang et al., 2018 | China | 2018 | NO_X_ per 10 ppb Increment | | CHD | 9 (3cohort/6case-control) | NA | | 1.01 (0.98, 1.04) |
| 13 | Zhang et al., 2018 | China | 2018 | NO_X_ per 10 ppb Increment | | VSD | 9 (3cohort/6case-control) | NA | | 1.03 (0.97, 1.10) |
| 14 | Hu et al.,2020 | China、Netherlands | 2020 | PM_10_ per 10 μg /m^3^（Continuous exposure） | | COA | 7 (2 cohort/5 case-control) | 802 | | 0.98 (0.86, 1.13) |
| 15 | Hu et al.,2020 | China、Netherlands | 2020 | PM_10_ per 10 μg /m^3^（Continuous exposure） | | PDA | 5(3 cohort/2 case-control） | 625 | | 1.06 (0.95, 1.19) |
| 16 | Hu et al.,2020 | China、Netherlands | 2020 | PM_10_ per 10 μg /m^3^（Continuous exposure） | | TOF | 11(3 cohort/8 case-control） | 983 | | 1.01 (0.94, 1.09) |
| 17 | Hu et al.,2020 | China、Netherlands | 2020 | PM_10_ per 10μg /m^3^（Continuous exposure） | | VSD | 14(4 cohort/10 case-control） | 5638 | | 1.00 (0.97, 1.03) |
| 18 | Yuet al., 2021 | China | 2021 | PM_10_ per 10μg/m^3^ | | ASD | 11(4 cohort, 7case-control) | 2148 | | 1.08 (0.99, 1.18) |
| 19 | Yuet al., 2021 | China | 2021 | PM_10_ per 10μg/m^3^ | | PVS | 6(1cohort, 5case-control) | 1186 | | 0.98 (0.89, 1.07) |
| 20 | Hu et al.,2020 | China、Netherlands | 2020 | PM_2.5_ per 10μg /m^3^（Continuous exposure） | | ASD | 7(2 cohort/5 case-control） | 2184 | | 1.08 (0.87, 1.34) |
| 21 | Hu et al.,2020 | China、Netherlands | 2020 | PM_2.5_ per 10μg /m^3^（Continuous exposure） | | TOF | 8 (3 cohort/5 case-control） | 375 | | 1.12 (0.98, 1.28) |
| 22 | Yuet al., 2021 | China | 2021 | PM_2.5_ per 10μg/m^3^ | | COA | 5(2cohort, 3 case-control) | 1313 | | 1.04 (0.93, 1.16) |
| 23 | Yuet al., 2021 | China | 2021 | PM_2.5_ per 10μg/m^3^ | | CHD | 7(4 cohort, 3case-control) | 5621 | | 1.04 (0.95, 1.13) |
| 24 | Yuet al., 2021 | China | 2021 | PM_2.5_ per 10μg/m^3^ | | PVS | 4(1cohort, 3 case-control) | 730 | | 0.99 (0.87, 1.14) |
| 25 | Yuet al., 2021 | China | 2021 | PM_2.5_ per 10μg/m^3^ | | VSD | 8(4 cohort, 4 case-control) | 4631 | | 1.03 (0.91, 1.17) |
| 26 | Hu et al.,2020 | China、Netherlands | 2020 | SO_2_ per 1 ppb（Continuous exposure） | | ASD | 8(2cohort/6 case-control） | 1792 | | 0.94 (0.86, 1.03) |
| 27 | Hu et al.,2020 | China、Netherlands | 2020 | SO_2_ per 1 ppb（Continuous exposure） | | COA | 5(2cohort/3 case-control） | 685 | | 1.01 (0.96, 1.06) |
| 28 | Hu et al.,2020 | China、Netherlands | 2020 | SO_2_ per 1 ppb（Continuous exposure） | | PDA | 4(3cohort/1 case-control） | 645 | | 0.98 (0.95, 1.02) |
| 29 | Hu et al.,2020 | China、Netherlands | 2020 | SO_2_ per 1 ppb（Continuous exposure） | | TOF | 8(3cohort/5 case-control） | 919 | | 0.99 (0.93, 1.06) |
| 30 | Hu et al.,2020 | China、Netherlands | 2020 | SO_2_ per 1 ppb（Continuous exposure） | | VSD | 10(4cohort/6 case-control） | 5338 | | 0.97(0.91,1.03) |
| **Reproductive related and assistive technologies** | | | | | | | | | | |
| 31 | Giorgione et al.,2018 | Italy | 2018 | ICSI/IVF pregnancies | | major CHD and minor CHD | 3(cohort) | 13376 | | 1.13 (0.68, 1.86) |
| 32 | Giorgione et al.,2018 | Italy | 2018 | ICSI/IVF pregnancies | | major CHD | 3(cohort) | 6688 | | 0.62 (0.16, 2.39) |
| 33 | Giorgione et al.,2018 | Italy | 2018 | ICSI/IVF pregnancies | | minor CHD | 3(cohort) | 6688 | | 1.30 (0.66, 2.56) |
| 34 | Ou et al., 2018 | China | 2018 | ICSI vs. IVF | | CHD | 13(cohort) | 755 | | 1.09 (0.93,1.27) |
| 35 | Ou et al., 2018 | China | 2018 | ICSI vs. IVF | | PDA | 3 (cohort) | 53 | | 1.07 (0.57, 2.04) |
| 36 | Ou et al., 2018 | China | 2018 | ICSI vs. IVF | | ASD | 4(cohort) | 34 | | 0.97 (0.47, 2.20) |
| 37 | Ou et al., 2018 | China | 2018 | ICSI vs. IVF | | VSD | 5 (cohort) | 54 | | 1.02 (0.53, 1.96) |
| 38 | Ou et al., 2018 | China | 2018 | ICSI vs. IVF | | TOF | 3 (cohort) | 7 | | 1.07 (0.28, 4.15) |
| **Parental age and BMI** | | | | | | | | | | |
| 39 | Peng et al., 2019 | China | 2019 | Paternal age (< 20) | | CHD | 5 observational studies | 495 | | 1.06 (0.72,1.54) |
| 40 | Peng et al., 2019 | China | 2019 | Paternal age (20–24) | | CHD | 5 observational studies | 2978 | | 0.90 (0.80,1.02) |
| 41 | Peng et al., 2019 | China | 2019 | Paternal age (25–29) | | CHD | 6 observational studies | 5745 | | 0.90 (0.82,0.98) |
| 42 | Peng et al., 2019 | China | 2019 | Paternal age (30–34) | | CHD | 6 observational studies | | 4816 | 0.99 (0.90,1.08) |
| 43 | Taylor et al., 2021 | UK | 2021 | Paternal BMI | | CHD | 7(cohort) | 1340 | | 1.01 (0.99, 1.03) |
| 44 | Zhu et al., 2018 | China | 2018 | Underweight | | CHD | 4 (1 cohort/13 case-control) | NA | | 1.02（0.98,1.05） |
| 45 | Stothard et al., 2009 | UK | 2009 | Maternal Obese | | TGA | 3 (case-control ) | 182 | | 1.41 (0.97, 2.06) |
| 46 | Cai et al., 2014 | China | 2014 | Underweight | | HLHS | 4 observational studies | 379 | | 0.85(0.60, 1.19) |
| 47 | Cai et al., 2014 | China | 2014 | Underweight | | OFT defect | 3 observational studies | 1645 | | 1.08 (0.68, 1.72) |
| 48 | Cai et al., 2014 | China | 2014 | Underweight | | ASD | 5 observational studies | 1983 | | 1.11 (0.85, 1.45) |
| 49 | Cai et al., 2014 | China | 2014 | Overweight | | ASD | 4 observational studies | 2382 | | 1.11 (0.94, 1.32) |
| 50 | Cai et al., 2014 | China | 2014 | Underweight | | TOF | 6 observational studies | 697 | | 1.01 (0.80, 1.27) |
| 51 | Cai et al., 2014 | China | 2014 | Overweight | | TOF | 5 observational studies | 948 | | 1.15 (0.97, 1.37) |
| 52 | Cai et al., 2014 | China | 2014 | Moderate obesity | | TOF | 3 observational studies | 703 | | 1.15 (0.94, 1.40) |
| 53 | Cai et al., 2014 | China | 2014 | Underweight | | CTD | 3 observational studies | 1078 | | 1.06 (0.88, 1.29) |
| 54 | Cai et al., 2014 | China | 2014 | Obesity | | VSD | 4 observational studies | 5230 | | 1.05 (0.94, 1.17) |
| 55 | Cai et al., 2014 | China | 2014 | Underweight | | COA | 4 observational studies | 449 | | 0.87 (0.64, 1.19) |
| 56 | Cai et al., 2014 | China | 2014 | Overweight | | COA | 4 observational studies | 575 | | 1.21 (0.95, 1.54) |
| 57 | Cai et al., 2014 | China | 2014 | Severe obesity | | COA | 3 observational studies | 424 | | 1.10 (0.70, 1.71) |
| 58 | Cai et al., 2014 | China | 2014 | Underweight | | TGA | 6 observational studies | 653 | | 0.90 (0.62, 1.31) |
| 59 | Cai et al., 2014 | China | 2014 | Overweight | | TGA | 5 observational studies | 724 | | 0.93 (0.78, 1.10) |
| 60 | Cai et al., 2014 | China | 2014 | Moderate obesity | | TGA | 3 observational studies | 543 | | 1.04 (0.76, 1.44) |
| 61 | Cai et al., 2014 | China | 2014 | Severe obesity | | TGA | 3 observational studies | 485 | | 1.19 (0.63, 2.25) |
| 62 | Cai et al., 2014 | China | 2014 | Obesity | | TGA | 5 observational studies | 678 | | 1.01 (0.78, 1.31) |
| 63 | Cai et al., 2014 | China | 2014 | Overweight | | CTD | 3 observational studies | 1358 | | 1.07 (0.95, 1.20) |
| 64 | Cai et al., 2014 | China | 2014 | Underweight | | AVSD | 3 observational studies | 807 | | 0.83 (0.66, 1.03) |
| 65 | Cai et al., 2014 | China | 2014 | Overweight | | AVSD | 3 observational studies | 862 | | 0.90 (0.75, 1.07) |
| 66 | Cai et al., 2014 | China | 2014 | Moderate obesity | | AVSD | 3 observational studies | 824 | | 1.04 (0.85, 1.28) |
| 67 | Cai et al., 2014 | China | 2014 | Obesity | | AVSD | 3 observational studies | 861 | | 1.11 (0.93, 1.33) |
| 68 | Cai et al., 2014 | China | 2014 | Underweight | | VSD | 5 observational studies | 4936 | | 0.98 (0.90, 1.07) |
| 69 | Cai et al., 2014 | China | 2014 | Overweight | | VSD | 4 observational studies | 5509 | | 0.99 (0.91, 1.09) |
| 70 | Cai et al., 2014 | China | 2014 | Moderate obesity | | VSD | 3 observational studies | 4972 | | 1.01 (0.90, 1.14) |
| **Parental life habits, working and dwelling environment** | | | | | | | | | | |
| 71 | Yue.et al.,2020 | China | 2020 | Coffee | | CHD | 10 observational studies | NA | | 1.03 (0.90, 1.19) |
| 72 | Yue.et al.,2020 | China | 2020 | Tea | | CHD | 3 observational studies | NA | | 1.38 (0.91, 2.11) |
| 73 | Peng et al.,2019 | China | 2019 | Paternal Smoking (1–9 cigarette/day) | | CHD | 4 observational studies | 434 | | 1.19 (0.82–1.71) |
| 74 | Zhao et al., 2019 | China | 2020 | Maternal active smoking | | AVSD | 8 observational studies | NA | | 1.27 (0.98,1.64) |
| 75 | Zhao et al., 2019 | China | 2020 | Maternal active smoking | | CTD | 6 observational studies | NA | | 1.12 (0.93,1.33) |
| 76 | Zhao et al., 2019 | China | 2020 | Maternal active smoking | | LVOTO | 5 observational studies | NA | | 0.97 (0.82,1.15) |
| 77 | Zhao et al., 2019 | China | 2020 | Maternal active smoking | | Septal defect | 4 observational studies | NA | | 1.19 (0.99,1.42) |
| 78 | Zhao et al., 2019 | China | 2020 | Maternal active smoking | | TGA | 7 observational studies | NA | | 1.14 (0.90,1.44) |
| 79 | Zhao et al., 2019 | China | 2020 | Maternal active smoking | | TOF | 6 observational studies | NA | | 1.00 (0.85,1.17) |
| 80 | Zhao et al., 2019 | China | 2020 | Maternal active smoking | | VSD | 12 observational studies | NA | | 1.14 (0.97,1.33) |
| 81 | Zhang et al., 2019 | China | 2019 | Maternal alcohol consumption | | VSD | 11 observational studies | NA | | 1.11 (0.98, 1.25) |
| 82 | Zhang et al., 2019 | China | 2019 | Maternal alcohol consumption | | ASD | 7 observational studies | NA | | 1.02 (0.83, 1.25) |
| 83 | Zhang et al., 2019 | China | 2019 | Maternal alcohol consumption | | AVSD | 3 observational studies | NA | | 1.00 (0.73, 1.37) |
| 84 | Zhang et al., 2019 | China | 2019 | Maternal alcohol consumption | | TGA | 5 observational studies | NA | | 1.11 (0.94, 1.31) |
| 85 | Zhang et al., 2019 | China | 2019 | Maternal alcohol consumption | | PVS | 3 observational studies | NA | | 0.87 (0.74, 1.01) |
| 86 | Zhang et al., 2019 | China | 2019 | Paternal alcohol consumption | | VSD | 6 observational studies | NA | | 1.35 (0.99, 1.84) |
| 87 | Zhang et al., 2019 | China | 2019 | Paternal alcohol consumption | | ASD | 3 observational studies | NA | | 2.60 (0.85, 7.96) |
| 88 | Spinder et al., 2019 | Netherlands | 2019 | Pesticides (Maternal occupational exposure) | | CHD | 5(case-control) | 4742 | | 0.81 (0.54, 1.21) |
| 89 | Spinder et al., 2019 | Netherlands | 2019 | Metals (Maternal occupational exposure) | | CHD | 3(case-control) | 1185 | | 1.83 (0.65, 5.20) |
| 90 | Zhou et al., 2016 | China | 2016 | Living close to the road during pregnancy | | CHD | 3 observational studies | NA | | 1.36 (0.50, 3.71) |
| 91 | Huo et al.. 2013 | China | 2013 | Contact with pets during early pregnancy | | CHD | 3(case-control) | 284 | | 2.30 (0.31, 16.84) |
| **Maternal drug exposure** | | | | | | | | | | |
| 92 | Budani et al., 2021 | Italy | 2021 | Oral fluconazole exposure during the first trimester of pregnancy (high dose) | | CHD | 3 (cohort) | 13356 | | 3.13 (0.53, 18.61) |
| 93 | Kaplan et al., 2019 | Turkey/UK | 2019 | ondansetron use during pregnancy | | CHD | 5(cohort) | NA | | 1.26 (0.90, 1.77) |
| 94 | Fan et al., 2019 | UK | 2019 | Prenatal use of macrolides | | CHD | 4 observational studies | 523 | | 1.14 (0.80, 1.62) |
| 95 | Fan et al., 2019 | UK | 2019 | Prenatal use of macrolides | | AVSD | 3 observational studies | 308 | | 1.16 (0.83, 1.62) |
| 96 | Kozer et al., 2002 | Canada | 2002 | Aspirin | | CHD | 6(4 case-control/ 2 cohort)* | 2986 | | 1.01 (0.91, 1.12) |
| 97 | Nieuwenhuijsen et al., 2009 | Spain | 2009 | High vs. low chlorination by-products | | Major CHD | 8 observational studies | NA | | 1.16 (0.98,1.37) |
| 98 | Nieuwenhuijsen et al., 2009 | Spain | 2009 | Per 10 µg/L TTHM | | Major CHD | 5 observational studies | NA | | 0.99 (0.95,1.04) |
| 99 | Goldberg et al., 2015 | Canada | 2015 | Nitrofurantoin during early pregnancy | | CHD | 6(2 case-control / 4 cohort)* | 23620 | | 0.94 (0.69, 1.28) |
| 100 | Wolf et al., 2017 | Denmark | 2017 | Periconceptional multivitamin use | | CHD | 6 observational studies | 1377 | | 0.83 (0.70, 0.98) |
| 101 | Zhang et al., 2017 | China | 2017 | Use of selective serotonin-reuptake inhibitors in the first trimester | | VSD | 7 (cohort) | NA | | 1.15 (0.97,1.36) |
| 102 | Gao et al., 2018 | China | 2018 | SSRIs | | VSD | 8(cohort) | NA | | 1.10 (0.94, 1.29) |
| 103 | Gao et al., 2018 | China | 2018 | SSRIs | | LVOTD | 3(cohort) | NA | | 1.08 (0.8, 1.44) |
| 104 | Gao et al., 2018 | China | 2018 | Citalopram | | ASD | 4(cohort) | NA | | 1.31 (0.61, 2.80) |
| 105 | Gao et al., 2018 | China | 2018 | Citalopram | | VSD | 4(cohort) | NA | | 1.18 (0.91, 1.52) |
| 106 | Gao et al., 2018 | China | 2018 | Fluoxetine | | ASD | 4(cohort) | NA | | 1.62 (0.90, 2.92) |
| 107 | Gao et al., 2018 | China | 2018 | Fluoxetine | | VSD | 5(cohort) | NA | | 1.12 (0.77, 1.63) |
| 108 | Gao et al., 2018 | China | 2018 | Paroxetine | | Septal defects | 4(cohort) | NA | | 1.58 (0.90, 2.78) |
| 109 | Gao et al., 2018 | China | 2018 | Paroxetine | | ASD | 4(cohort) | NA | | 2.06 (0.92, 4.60) |
| 110 | Gao et al., 2018 | China | 2018 | Paroxetine | | VSD | 5(cohort) | NA | | 1.37 (0.90, 2.08) |
| 111 | Gao et al., 2018 | China | 2018 | Sertraline | | VSD | 5(cohort) | NA | | 1.21 (0.65, 2.22) |
| 112 | Gao et al., 2018 | China | 2018 | Escitalopram | | CHD | 6(cohort) | NA | | 0.87 (0.69, 1.10) |
| 113 | De Vries et al., 2021 | Australia | 2021 | TCA | | CHD | 5 (4 cohort/1 case-control) | 10197 | | 1.02 (0.82, 1.25) |
| 114 | De Vries et al., 2021 | Australia | 2021 | Venlafaxine | | CHD | 4 (3 cohort/1 case-control)* | 28467 | | 1.30 (0.99, 1.71) |
| 115 | Huo et al., 2013 | China | 2013 | NSAIDs | | CHD | 4(case-control) | 853 | | 2.52(0.68, 9.28) |
| 116 | Bracken et al. | USA | 1989 | Oral contraceptive | | CHD | 8(cohort) | 713 | | 1.06 (0.72, 1.56) |
| **Maternal disease** | | | | | | | | | | |
| 117 | Ye et al., 2019 | China | 2019 | Herpes virus (fixed-effect model) | | CHD | 6(case-control) | NA | | 1.00 (0.60,1.64) |
| 118 | Ye et al., 2019 | China | 2019 | Hepatitis B virus (fixed-effect model) | | CHD | 4(case-control) | NA | | 2.07 (0.64,6.69) |
| 119 | Ye et al., 2019 | China | 2019 | Coxsackie virus (fixed-effect model) | | CHD | 3(case-control) | NA | | 1.32 (0.96,1.82) |
| 120 | Ye et al., 2019 | China | 2019 | Other virus (fixed-effect model) | | CHD | 3(case-control) | NA | | 1.27 (0.72,2.24) |
| 121 | Ye et al., 2019 | China | 2019 | Herpes virus (random-effect model) | | CHD | 6(case-control) | NA | | 1.10 (0.50,2.41) |
| 122 | Ye et al., 2019 | China | 2019 | Cytomegalovirus (random-effect model) | | CHD | 4(case-control) | NA | | 5.14 (0.99,26.74) |
| 123 | Ye et al., 2019 | China | 2019 | Hepatitis B virus (random-effect model) | | CHD | 4(case-control) | NA | | 3.53 (0.42,29.93) |
| 124 | Ye et al., 2019 | China | 2019 | Coxsackie virus (random-effect model) | | CHD | 3(case-control) | NA | | 1.36 (0.82,2.27) |
| 125 | Ye et al., 2019 | China | 2019 | Other virus (random-effect model) | | CHD | 3(case-control) | NA | | 1.97 (0.40,9.57) |
| 126 | Fornaro et al., 2020 | Italy | 2020 | Exposure to lithium at any time during pregnancy compared with unexposed patients with bipolar disorder | | CHD | 4(cohort) | 1508 | | 1.59 (0.91,2.77) |
| 127 | Yang et al., 2021 | China | 2021 | Maternal fever | | TOF | 3(case-control) | 11304 | | 1.11 (0.94, 1.32) |
| 128 | Yang et al., 2021 | China | 2021 | Maternal fever | | HLHS | 4(case-control) | 13665 | | 1.17 (0.94, 1.47) |
| 129 | Yang et al., 2021 | China | 2021 | Maternal fever | | VSD | 6(case-control) | 18479 | | 1.21 (0.76, 1.94) |
| 130 | Yang et al., 2021 | China | 2021 | Maternal fever | | LVOTO | 3(case-control) | 13071 | | 1.08 (0.52, 2.24) |
| 131 | Ma et al., 2017 | China | 2017 | Gestational hypertension | | LVOTO | 5 observational studies | NA | | 1.14 (0.93, 1.40) |
| 132 | Ma et al., 2017 | China | 2017 | Gestational hypertension | | RVOTO | 3 observational studies | NA | | 1.71(0.72, 4.03) |
| 133 | Chen et al., 2019 | China | 2019 | Maternal DM | | PVS | 4 observational studies | NA | | 1.65 (0.96, 2.85) |
| 134 | Chen et al., 2019 | China | 2019 | PGDM | | PVS | 3 observational studies | NA | | 1.75 (0.75, 4.10) |
| 135 | Chen et al., 2019 | China | 2019 | GDM | | TGA | 4 observational studies | NA | | 1.44 (0.94, 2.21) |
| 136 | Chen et al., 2019 | China | 2019 | GDM | | HLHS | 4 observational studies | NA | | 1.31(0.89, 1.92) |

**Abbreviation**: OR, Odds ratio; RR, relative risk; CI, Confidence interval; NA, Not available; IVF: In-vitro-fertilization; ICSI: Intracytoplasmic sperm injection; TTHM, Total trihalomethane; SSRI, Selective serotonin reuptake inhibitor; TCA, Tricyclic antidepressant; NSAIDs, Nonsteroidal anti-inflammatory drugs; DM, Diabetes mellitus; GDM, Gestational diabetes mellitus; PGDM, Pregestational diabetes mellitus; ASD, Atrial septal defect; TOF, Tetralogy of fallot; VSD, Ventricular septal defects; CTD, Conotruncal defects; COA, Coarctation of the aorta; PDA, Patent ductus arteriosus; PVS, Pulmonary valve stenosis; CHD, Congenital heart disease; TGA, Transposition of great arteries; HLHS, Hypoplastic left heart syndrome; OFT, outflow tract; AVSD, Atrioventricular septal defect; LVOTD, Left ventricular outflow tract obstruction; PVS, Pulmonary valve stenosis; RVOTD, Right ventricular outflow tract obstruction. * NA, the number can not be available from meta-analysis

**Supplementary Table S5 The characteristic of specific association which the number of included studies less than three (n=48)**

| **ID** | **Author** | **Country** | **Year of publication** | **Exposure** | **Outcome** | **Number of studies included** | **OR/RR** | **OR/ RR  (95% CI)** |
| --- | --- | --- | --- | --- | --- | --- | --- | --- |
| 1 | Yu et al.,2018 | China | 2018 | Eat more meat during pregnancy | CHD | 2 | OR | 0.74 (0.56, 0.98) |
| 2 | Yue et al.,2020 | China | 2020 | Caffeine consumption (Low) | CHD | 2 | OR | 1.20 (1.05, 1.36) |
| 3 | Yue et al.,2020 | China | 2020 | Caffeine consumption (Moderate) | CHD | 2 | OR | 1.32 (1.05, 1.65) |
| 4 | Yue et al.,2020 | China | 2020 | Soft drinks | CHD | 1 | OR | 1.71 (0.71, 4.11) |
| 5 | Stothard et al., 2009 | UK | 2009 | Maternal Overweight | Septal defects | 2 | OR | 1.15 (0.71,1.85) |
| 6 | Spinder et al., 2019 | Netherlands | 2019 | Glycol ethers (Maternal occupational exposure) | CHD | 2 | OR | 1.63 (0.94,2.84) |
| 7 | Zhang et al.,2019 | China | 2019 | Oral fluconazole during the first trimester of pregnancy | TOF | 2 | OR | 3.39(1.71,6.74) |
| 8 | Zhang et al.,2019 | China | 2019 | Oral fluconazole during the first trimester of pregnancy | ASD | 2 | OR | 1.29(0.91,1.82) |
| 9 | Gao et al., 2018 | China | 2018 | SSRIs | CTD | 2 | OR | 2.28 (0.88, 5.94) |
| 10 | Gao et al., 2018 | China | 2018 | Citalopram | RVOTD | 2 | OR | 1.59 (1.08, 2.35) |
| 11 | Gao et al., 2018 | China | 2018 | Citalopram | LVOTD | 2 | OR | 1.50 (0.98, 2.30) |
| 12 | Gao et al., 2018 | China | 2018 | Citalopram | CHD | 1 | OR | 2.09 (1.25, 3.50) |
| 13 | Gao et al., 2018 | China | 2018 | Fluoxetine | LVOTD | 2 | OR | 0.85 (0.41, 1.78) |
| 14 | Gao et al., 2018 | China | 2018 | Fluoxetine | CHD | 1 | OR | 1.72 (0.85, 3.48) |
| 15 | Gao et al., 2018 | China | 2018 | Paroxetine | LVOTD | - | - | - |
| 16 | Gao et al., 2018 | China | 2018 | Paroxetine | CTD | 1 | OR | 1.59 (0.51, 4.95) |
| 17 | Gao et al., 2018 | China | 2018 | Sertraline | RVOTD | 2 | OR | 1.18 (0.82, 1.68) |
| 18 | Gao et al., 2018 | China | 2018 | Sertraline | LVOTD | 1 | OR | 0.82 (0.37, 1.83) |
| 19 | Gao et al., 2018 | China | 2018 | Sertraline | CHD | 1 | OR | 2.12 (0.88, 5.11) |
| 20 | Gao et al., 2018 | China | 2018 | Escitalopram | Septal defects | 2 | OR | 2.15 (0.59, 7.86) |
| 21 | Gao et al., 2018 | China | 2018 | Escitalopram | ASD | 2 | OR | 1.43 (0.79, 2.59) |
| 22 | Gao et al., 2018 | China | 2018 | Escitalopram | VSD | 2 | OR | 1.04 (0.56, 1.93) |
| 23 | Gao et al., 2018 | China | 2018 | Escitalopram | RVOTD | - | - | - |
| 24 | Gao et al., 2018 | China | 2018 | Escitalopram | LVOTD | 1 | OR | 2.21 (0.31, 15.86) |
| 25 | Gao et al., 2018 | China | 2018 | Escitalopram | CHD | 1 | OR | 0.55 (0.18, 1.70) |
| 26 | Gao et al., 2018 | China | 2018 | Fluvoxamine | CHD | 1 | OR | 0.56 (0.14, 2.25) |
| 27 | Gao et al., 2018 | China | 2018 | Fluvoxamine | VSD | 1 | OR | 0.42 (0.06, 3.00) |
| 28 | Vahedian-Azimi et al., 2021 | Iran | 2021 | Statin exposure | CHD | 2 | RR | 2.53 (0.81, 7.93) |
| 29 | Ma et al., 2017 | China | 2017 | Gestational hypertension | TOF | 2 | OR | 1.60 (1.09, 2.50) |
| 30 | Ma et al., 2017 | China | 2017 | Gestational hypertension | PVS | 1 | OR | 2.60 (1.30, 5.40) |
| 31 | Chen et al., 2019 | China | 2019 | Maternal DM | DORV | 2 | OR | 10.89 (8.77, 13.53) |
| 32 | Chen et al., 2019 | China | 2019 | Maternal DM | CHD | 1 | OR | 1.75 (0.86, 3.57) |
| 33 | Chen et al., 2019 | China | 2019 | Maternal DM | TVS | 2 | OR | 2.44 (1.77, 3.37) |
| 34 | Chen et al., 2019 | China | 2019 | PGDM | DORV | 2 | OR | 11.51 (9.24, 14.32) |
| 35 | Chen et al., 2019 | China | 2019 | PGDM | CTD | 1 | OR | 6.28 (1.06, 37.21) |
| 36 | Chen et al., 2019 | China | 2019 | PGDM | TVS | 2 | OR | 7.61 (4.19, 13.84) |
| 37 | Chen et al., 2019 | China | 2019 | GDM | Heterotaxia | 2 | OR | 0.72(0.17, 3.00) |
| 38 | Chen et al., 2019 | China | 2019 | GDM | AVSD | 2 | OR | 1.55(1.13, 2.14) |
| 39 | Chen et al., 2019 | China | 2019 | GDM | PDA | 1 | OR | 2.62(0.92, 7.46) |
| 40 | Chen et al., 2019 | China | 2019 | GDM | TA | 2 | OR | 2.38(1.07, 5.26) |
| 41 | Chen et al., 2019 | China | 2019 | GDM | DORV | 1 | OR | 2.26(0.53, 9.64) |
| 42 | Chen et al., 2019 | China | 2019 | GDM | APVR | 2 | OR | 1.12(0.18, 7.08) |
| 43 | Chen et al., 2019 | China | 2019 | GDM | RVOTD | 2 | OR | 1.36(1.14, 1.63) |
| 44 | Chen et al., 2019 | China | 2019 | GDM | PVS | 2 | OR | 1.58(0.78, 3.19) |
| 45 | Chen et al., 2019 | China | 2019 | GDM | CTD | 2 | OR | 1.51(0.82, 2.79) |
| 46 | Chen et al., 2019 | China | 2019 | GDM | TA | 1 | OR | 1.97(1.38, 2.81) |
| 47 | Chen et al., 2019 | China | 2019 | GDM | Septal defects | 2 | OR | 1.57(1.16, 2.13) |
| 48 | Chen et al., 2019 | China | 2019 | GDM | Single ventricle | 1 | OR | 1.37(0.69, 2.72) |

**Abbreviation:** OR, Odds ratio; RR, relative risk; CHD, Congenital heart disease; TOF, Tetralogy of fallot; ASD, Atrial septal defect; RVOTD, Right ventricular outflow tract obstruction; LVOTD, Left ventricular outflow tract obstruction; VSD, Ventricular septal defects; PVS, Pulmonary valve stenosis; DORV, Double outlet of the right ventricle; TVS, Tricuspid valve stenosis; AVSD, Atrioventricular septal defect; PDA, Patent ductus arteriosus; TA, Truncus arteriosus; APVR , Anomalous pulmonary venous return; TA, truncus arteriosus.

**Supplementary Table S6 AMSTAR 2 quality assessment of 56 included systematic review and meta-analyses**

|  |  | **AMSTAR 2 items ^a, c^** | | | | | | | | | | | | | | | | |
| --- | --- | --- | --- | --- | --- | --- | --- | --- | --- | --- | --- | --- | --- | --- | --- | --- | --- | --- |
| **ID** | **Author, Year**  **[Reference]** | **1** | **2** ^b^ | **3** | **4** ^b^ | **5** | **6** | **7** ^b^ | **8** | **9** ^b^ | **10** | **11** ^b^ | **12** | **13** ^b^ | **14** | **15** ^b^ | **16** | **Overall rating** (based on critical domains)**^d^** |
| 1 | Spinder et al., 2019 | Y | pY | Y | pY | Y | Y | N | pY | pY | N | Y | N | Y | Y | Y | Y | Low |
| 2 | Zhang et al., 2017 | Y | pY | Y | pY | Y | Y | N | pY | N | N | Y | N | Y | Y | Y | Y | Critically low |
| 3 | Zhao et al., 2020 | Y | pY | Y | pY | Y | Y | N | pY | Y | N | Y | Y | Y | Y | Y | Y | Low |
| 4 | Lutejin et al., 2014 | Y | pY | Y | pY | Y | Y | N | pY | pY | N | Y | Y | Y | Y | Y | Y | Low |
| 5 | Ye et al., 2019 | Y | pY | Y | pY | Y | Y | N | pY | pY | N | Y | Y | Y | Y | Y | N | Critically low |
| 6 | Hoang et al., 2017 | Y | pY | Y | pY | N | N | N | pY | N | N | Y | Y | Y | Y | N | Y | Critically low |
| 7 | Cai et al., 2014 | Y | pY | Y | pY | Y | Y | N | pY | N | N | Y | N | Y | Y | Y | Y | Critically low |
| 8 | Zhu et al., 2018 | Y | pY | Y | pY | Y | Y | N | pY | pY | N | Y | N | Y | Y | Y | Y | Low |
| 9 | Feng et al., 2014 | Y | pY | Y | pY | Y | Y | N | pY | pY | N | Y | N | Y | Y | Y | Y | Low |
| 10 | Tanoshima,et al., 2015 | Y | pY | Y | pY | Y | Y | N | pY | pY | N | Y | N | Y | Y | Y | Y | Low |
| 11 | Gao et al.2018 | Y | PY | Y | PY | Y | Y | PY | Y | PY | N | Y | Y | Y | Y | Y | Y | Moderate |
| 12 | Feng et al., 2015 | Y | pY | Y | pY | Y | Y | N | pY | pY | N | Y | N | Y | Y | Y | Y | Low |
| 13 | Xu et al., 2016 | Y | pY | Y | pY | Y | Y | N | pY | pY | N | Y | N | N | Y | Y | Y | Critically low |
| 14 | Yu et al., 2014 | Y | pY | Y | pY | Y | Y | N | pY | N | N | Y | N | Y | Y | Y | Y | Critically low |
| 15 | Peng et al., 2019 | Y | pY | Y | pY | Y | Y | N | pY | Y | N | Y | N | N | Y | Y | Y | Critically low |
| 16 | Kozer et al., 2002 | Y | pY | Y | pY | Y | Y | N | N | N | N | Y | N | Y | Y | N | Y | Critically low |
| 17 | Huo et al.2013 | Y | PY | Y | PY | Y | Y | N | PY | N | N | Y | N | N | Y | Y | N | Critically low |
| 18 | Nieuwenhuijsen et al., 2009 | Y | pY | Y | pY | N | N | N | pY | N | N | Y | N | N | Y | Y | N | Critically low |
| 19 | Stothard et al., 2009 | Y | pY | Y | pY | N | N | Y | pY | N | N | Y | N | N | Y | Y | N | Critically low |
| 20 | Wang et al., 2015 | Y | pY | Y | pY | N | N | N | N | pY | N | Y | N | N | Y | Y | N | Critically low |
| 21 | Zhang et al., 2019 | Y | pY | Y | PY | Y | Y | Y | PY | PY | N | Y | N | Y | Y | Y | Y | low |
| 22 | Kaplan et al.2019 | Y | pY | Y | PY | N | Y | N | PY | PY | N | Y | Y | Y | Y | N | Y | Critically low |
| 23 | Zheng et al., 2018 | Y | pY | Y | PY | N | Y | N | PY | PY | N | Y | Y | Y | Y | Y | Y | Critically low |
| 24 | Zhang et al., 2018 | Y | pY | Y | PY | N | Y | N | PY | PY | N | Y | Y | Y | Y | Y | Y | low |
| 25 | Ma et al., 2017 | Y | pY | Y | PY | Y | Y | N | N | N | N | Y | Y | Y | Y | Y | N | Critically low |
| 26 | Grigoriadis et al., 2014 | Y | pY | Y | PY | N | Y | PY | PY | PY | N | Y | Y | Y | Y | Y | N | Moderate |
| 27 | Fang et al,2020 | Y | PY | Y | PY | Y | Y | PY | PY | Py | N | Y | Y | Y | Y | Y | Y | Moderate |
| 28 | Taylor et al., 2021 | Y | PY | N | N | N | N | N | N | N | N | N | N | N | N | N | N | Critically low |
| 29 | Dong et al., 2013 | Y | PY | Y | PY | N | N | N | PY | N | N | Y | N | N | Y | Y | N | Critically low |
| 30 | Zhou et al., 2016 | Y | PY | Y | PY | Y | Y | N | N | N | N | Y | N | N | Y | Y | N | Critically low |
| 31 | Budani et al.,2021 | Y | PY | Y | N | N | N | N | PY | N | N | Y | Y | Y | Y | N | Y | Low |
| 32 | Picot et al.,2020 | Y | PY | Y | PY | Y | Y | N | Y | Y | N | Y | Y | Y | Y | Y | Y | Low |
| 33 | Fan et al.,2019 | Y | PY | Y | PY | Y | Y | PY | N | Y | N | Y | Y | Y | Y | Y | Y | Moderate |
| 34 | Carl et al.,2018 | Y | PY | Y | PY | Y | Y | PY | Y | Y | N | Y | Y | Y | Y | Y | Y | Moderate |
| 35 | Goldberg et al., 2015 | Y | PY | Y | PY | Y | Y | PY | N | N | N | Y | N | N | Y | N | N | Critically low |
| 36 | Wolf et al., 2017 | Y | PY | Y | Y | Y | Y | Y | Y | PY | N | Y | Y | Y | Y | Y | N | Moderate |
| 37 | De Vries et al. ,2021 | Y | PY | Y | PY | Y | Y | N | Y | Y | N | Y | Y | Y | Y | Y | Y | Low |
| 38 | Wu et al.,2021 | Y | PY | Y | PY | Y | Y | N | Y | PY | N | Y | Y | N | Y | Y | N | Critically low |
| 39 | Vahedian-Azimi et al.,2021 | Y | PY | Y | PY | Y | Y | PY | Y | Y | N | Y | Y | N | Y | Y | N | Low |
| 40 | Rahimi et al., 2021 | Y | PY | Y | PY | Y | Y | N | Y | Y | N | Y | Y | N | Y | Y | Y | Critically low |
| 41 | Fornaro et al.,2020 | Y | PY | Y | PY | Y | Y | PY | Y | Y | N | Y | Y | N | Y | Y | Y | Low |
| 42 | Yang et al.,2021 | Y | Y | Y | Y | Y | Y | PY | PY | Y | N | Y | Y | N | Y | Y | Y | Low |
| 43 | Zhu et al.,2020 | Y | PY | Y | PY | N | N | N | pY | N | N | Y | N | N | Y | N | N | Critically low |
| 44 | Jiang et al.,2019 | Y | PY | Y | PY | Y | Y | N | PY | N | N | Y | N | N | Y | Y | N | Critically low |
| 45 | Chen et al.,2019 | Y | PY | Y | PY | Y | Y | N | Y | Y | N | Y | Y | Y | Y | Y | Y | Low |
| 46 | Wu et al.,2021 | Y | PY | Y | PY | Y | Y | N | Y | PY | N | Y | Y | N | Y | Y | N | Low |
| 47 | Yu et al.,2021 | Y | PY | Y | PY | Y | Y | N | Y | PY | N | Y | Y | Y | Y | Y | Y | Critically low |
| 48 | Li et al.,2017 | Y | PY | Y | PY | N | N | N | PY | N | N | Y | N | N | Y | N | N | Critically low |
| 49 | Gijtenbeek et al.,2019 | Y | PY | Y | PY | Y | Y | N | Y | PY | N | Y | Y | N | Y | Y | N | Critically low |
| 50 | Hu et al.,2020 | Y | PY | Y | PY | Y | Y | PY | Y | PY | N | Y | Y | Y | Y | Y | Y | Moderate |
| 51 | Ou et al., 2018 | Y | PY | Y | PY | Y | Y | N | PY | PY | N | Y | N | N | Y | Y | N | Critically low |
| 52 | Ma et al., 2014 | Y | PY | Y | PY | Y | Y | N | PY | PY | N | Y | N | N | Y | Y | N | Critically low |
| 53 | Yan et al.,2021 | Y | PY | Y | PY | Y | Y | PY | Y | PY | N | Y | Y | Y | Y | Y | Y | Moderate |
| 54 | Zhang et al.,2020 | Y | PY | Y | PY | Y | Y | N | N | PY | N | Y | Y | N | Y | Y | Y | Moderate |
| 55 | Picot et al.2020 | Y | PY | Y | N | N | N | N | N | N | N | N | N | N | N | N | N | Critically low |
| 56 | Brcken et al.,1990 | Y | PY | Y | N | N | N | N | PY | PY | N | Y | N | N | N | N | N | Critically low |

^a^ Y: Yes; PY: Partly yes; N: No.

^b^ Critical Domains

^c^ AMSTAR 2 items:

1. **Did the research questions and inclusion criteria for the review include the components of PICO (Population, Intervention, Comparator group, Outcome)?** YES/NO. For yes, must have all four.
2. **Did the report of the review contain an explicit statement that the review methods were established prior to the conduct of the review and did the report justify any significant deviations from the protocol?** YES, PARTIAL YES, NO. For Partial YES: the authors state that they had a written protocol or guide that included ALL the following (review question(s), a search strategy, inclusion/exclusion criteria, a risk of bias assessment). For YES: as for partial yes, plus the protocol should be registered and should also have specified: a meta-analysis/synthesis plan, if appropriate, and a plan for investigating causes of heterogeneity, justification for any deviations from the protocol.
3. **Did the review authors explain their selection of the study designs for inclusion in the review?** YES/NO. For YES, the review should satisfy one of the following: explanation for including only RCTs, or explanation for including only NRSI, or explanation for including both RCTs and NRSI.
4. **Did the review authors use a comprehensive literature search strategy?** YES, PARTIAL YES, NO. for PARTIAL YES must have all of the following: searched at least 2 databases (relevant to research question), provided key word and/or search strategy, justified publication restrictions (e.g., Language). For YES should also have all of the following: searched the reference lists/biographies of included studies, searched trial/study registries, included/consulted content experts in the field, searched for grey literature where relevant, conducted search within 24 months of completion of the review.
5. **Did the review authors perform study selection in duplicate?** YES/NO. for YES, either ONE of the following: at least two reviewers independently agreed on selection of eligible studies and achieved consensus on which studies to include OR two reviewers selected a sample of eligible studies and achieved good agreement (at least 80 per cent) with the remainder selected by one reviewer.
6. **Did the review authors perform data extraction in duplicate?** YES/NO. For YES, either one of the following: at least two reviewers achieved consensus on which data to extract from included studies OR two reviewers extracted data from a sample of eligible studies and achieved good agreement (at least 80 per cent) with the remainder extracted by one reviewer.
7. **Did the review authors provide a list of excluded studies to justify the exclusions?** YES, PARTIAL YES, NO. FOR partial yes must provide a list of all potentially relevant studies that were read in full text form but excluded from the review. For YES must also have justified the exclusion from the review of each potentially relevant study.
8. **Did the review authors describe the included studies in adequate detail?** YES, PARTIAL YES, NO. For PARTIAL YES, must describe all of the following: populations, interventions, comparators, outcomes, research designs. For YES should also have all the following: described populations in detail, described intervention and comparator in detail (including doses where relevant), described study setting, timeframe or follow-up.
9. **Did the review authors use a satisfactory technique for assessing the risk of bias (RoB) in individual studies that were included in the review? For RCTs**: YES, PARTIAL YES, NO, INCLUDES ONLY NRSI. For PARTIAL YES must have assessed RoB from unconcealed allocation and lack of blinding of patients and assessors when assessing outcomes (unnecessary for objective outcomes such as all-cause mortality) (for YES must also have assessed RoB from allocation sequence that was not truly random and selection of the reported result from among multiple measurements or analyses of a specified outcome. **For NRSI** (Non-Randomized Studies of Intervention)**:** YES, PARTIAL YES, NO, INCLUDES ONLY RCTs. For PARTIAL YES must have assessed RoB from confounding and from selection bias. For YES, must also have assessed methods used to ascertain exposures and outcomes, and selection of the reported results from among multiple measurements or analyses of a specified outcome.
10. **Did the review authors report on the sources of funding for the studies included in the review?** YES/NO. For YES: must have reported on the sources of funding for individual studies included in the review. Note: reporting that the reviewers looked for this information, but it was not reported by study authors also qualifies
11. **If meta-analysis was performed, did the review authors use appropriate methods for statistical combination of results? For RCTs:** YES, NO, NO META-ANALYSIS. For YES: the authors justified combining the data in a meta-analysis and they used an appropriate weighted technique to combine study results and adjusted for heterogeneity if present and investigated the causes of heterogeneity. **For NRSI:** YES, NO, NO META-ANALYSIS CONDUCTED. For YES: the authors justified combining the data in a meta-analysis and they used an appropriate weighted technique to combine study results, adjusting for heterogeneity if present, and they statistically combined effects estimates from NRSI that were adjusted for confounding, rather than combining raw data, or justified combining raw data when adjusted effect estimates were not available, and they reported separate summary estimates for RCTs and NRSI separately when both were included in the review.
12. **If meta-analysis was performed, did the review authors assess the potential impact of RoB in individual studies on the results of the meta-analysis or other evidence synthesis?** YES, NO, NO META-ANALYSIS INCLUDED. For YES: included only low risk of bias RCTs or, if the pooled estimate was based on RCTs and/or NRSI at variable RoB, the authors performed analysis ton investigate possible impact of RoB on summary estimates of effect.
13. **Did the review authors account for RoB in individual studies when interpreting/discussing the results of the review?** YES/NO. for YES: included only low risk of bias RCTs or, if RCTs with moderate or high RoB, or NRSI were included, the review provided a discussion of the key impact of RoB on the results
14. **Did the review authors provide a satisfactory explanation for, and discussion of, any heterogeneity observed in the results of the review?** YES/NO. For Yes: there was no significant heterogeneity in the results OR if heterogeneity was present the authors performed an investigation of sources of any heterogeneity in the results and discussed the impact of this on the results of the review
15. **If they performed quantitative synthesis did the review authors carry out an adequate investigation of publication bias (small study bias) and discuss its likely impact on the results of the review?** YES, NO, NO META-ANALYSIS CONDUCTED. For YES: performed graphical statistical tests for publication bias and discussed the likelihood and magnitude of impact of publication bias
16. **Did the review authors report any potential sources of conflict of interest, including any funding they received for conducting the review?** YES/NO. For Yes: the authors reported no competing interests OR the authors described their funding sources and how they managed potential conflicts of interest.

**^d^** Rating overall confidence in the results of the review:

HIGH: *no on one non-critical weakness*: the systematic review provides an accurate and comprehensive summary of the results of the available studies that address the question of interest

MODERATE: *more than one noncritical weakness* (multiple non-critical weaknesses may diminish confidence in the review and it may be appropriate to move the overall appraisal down from moderate to low confidence): the systematic review has more than one weakness but no critical flaws. It may provide an accurate summary of the results of the available studies that were included in the review

LOW: *one critical flaw with or without non-critical weaknesses*: the review has a critical flaw and may not provide an accurate and comprehensive summary of the available studies that address the question of interest

CRITICALLY LOW: *more than one critical flaw with or without non-critical weaknesses*: the review has more than one critical flaw and should not be relied on to provide an accurate and comprehensive summary of the available studies.

**Supplementary Table S7 Sensitivity analysis of eligible associations for specific factor and CHD (only included cohort studies)**

| **Factor*** | **Studies** | **Cases** | **Egger test** | **Random-effect** | **95%PI** | **Heterogeneity** | **Excess significance bias** | **Fixed-effect** | **Random-effect** | **Grade^#^** |
| --- | --- | --- | --- | --- | --- | --- | --- | --- | --- | --- |
|  |  |  | ***p* value** | **summary effect size(95%CI)** |  | ***I^2^*(%)** | ***p* value** | ***p* value** | ***p* value** |  |
| **$Reproductive related and assistive technologies** | | | | | | | | | | |
| Maternal parity | 3 | 22488 | 0.01 | 1.21(1.06,1.37) | (0.24,6.12) | 88.89 | 1.00 | 3.13E-48 | 4.40E-03 | IV |
| ICSI vs IVF (in fresh transplantation cycle) | 3 | 72 | 0.08 | 2.07(1.28,3.36) | (0.09,47.47) | 0.00 | 1.00 | 3.05E-03 | 3.05E-03 | IV |
| Singleton IVF/ICSI | 5 | 2159 | 0.11 | 1.56(1.21,2.00) | (0.79,3.08) | 35.18 | 0.66 | 3.23E-05 | 5.89E-04 | III |
| ICSI/IVF pregnancies | 8 | 1047 | 0.61 | 1.45(1.21,1.73) | (0.94,2.23) | 43.62 | 0.57 | 5.78E-08 | 4.69E-05 | III |
| MC twins with TTTS vs. MC twins without TTTS | 2 | 149 | — | 2.31(1.53,3.49) | — | 0.00 | 0.75 | 6.23E-05 | 6.23E-05 | IV |
| MC twins without TTTS | 2 | 108 | — | 3.78(2.54,5.64) | — | 0.00 | 0.99 | 2.12E-12 | 2.12E-12 | IV |
| MC twins with TTTS | 3 | 118 | 0.63 | 13.54(8.35,21.94) | (0.59,309.58) | 0.00 | 0.96 | 3.80E-26 | 3.80E-26 | IV |
| MC twins | 3 | 115 | 0.29 | 6.42(4.11,10.03) | (0.36,115.60) | 0.00 | 0.69 | 2.89E-16 | 2.89E-16 | IV |
| **Parental Demographic data** | | | | | | | | | | |
| Maternal obesity | 5 | 39133 | 0.64 | 1.27(1.14,1.43) | (0.90,1.81) | 70.65 | 0.86 | 9.39E-30 | 3.03E-05 | III |
| Maternal overweight | 3 | 18279 | 0.78 | 1.01(0.83,1.23) | (0.10,10.01) | 68.91 | 0.27 | 1.31E-03 | 8.97E-01 | NS |
| Paternal age (≥40 years) | 4 | 3250 | 0.71 | 1.43(0.90,2.25) | (0.18,11.44) | 97.14 | 0.25 | 2.07E-08 | 1.27E-01 | NS |
| **Parental Life habits and work exposure** | | | | | | | | | | |
| Maternal educational attainment | 2 | 6165 | — | 1.21(1.06,1.37) | — | 57.04 | 0.6 | 1.37E-34 | 4.49E-03 | IV |
| Maternal passive smoking | 3 | 417 | 0.90 | 1.27(0.48,3.35) | (0.00,120196.62) | 86.04 | 0.85 | 1.08E-01 | 6.24E-01 | NS |
| Maternal active smoking | 14 | 24649 | 0.32 | 1.16(1.00,1.34) | (0.68,1.97) | 84.22 | 0.47 | 4.28E-05 | 5.12E-02 | NS |
| Lithium exposure (in the first trimester compared with patients with bipolar disordered） | 3 | 59 | 0.39 | 1.96(1.13,3.39) | (0.06,69.51) | 0.00 | 1.00 | 1.69E-02 | 1.69E-02 | IV |
| Lithium exposure (in the first trimester compared with general population） | 4 | 15293 | 0.41 | 4.90(1.72,13.96) | (0.07,322.1) | 69.14 | 0.08 | 2.70E-07 | 2.95E-03 | IV |
| Lithium exposure (in the first trimester compared with unexposed women） | 3 | 15314 | 0.28 | 4.56(1.59,13.12) | (0.00,627860.53) | 71.78 | 0.06 | 1.41E-06 | 4.89E-03 | IV |
| **Maternal drug exposure** | | | | | | | | | | |
| β-blockers (in the first trimester) | 4 | 12830 | 0.79 | 1.42(0.76,2.68) | (0.1,20.15) | 81.15 | 0.99 | 1.17E-03 | 2.71E-01 | NS |
| Sertraline | 13 | 74598 | 0.26 | 1.44(1.09,1.91) | (0.59,3.53) | 63.82 | 0.02 | 4.33E-06 | 9.22E-03 | IV |
| SNRI | 3 | 24655 | 0.61 | 1.59(1.34,1.88) | (0.53,4.75) | 0.00 | 0.79 | 7.53E-08 | 7.53E-08 | II |
| SSRIs | 14 | 42236 | 0.95 | 1.27(1.19,1.34) | (1.19,1.35) | 36.88 | 0.34 | 3.69E-15 | 3.85E-15 | I |
| Any antidepressant (in the first trimester) | 15 | 60215 | 0.80 | 1.27(1.14,1.42) | (0.94,1.72) | 50.06 | 0.54 | 8.95E-20 | 1.35E-05 | III |
| Fluoxetine | 14 | 74523 | 0.33 | 1.30(1.13,1.50) | (0.98,1.72) | 28.25 | 0.19 | 3.90E-06 | 2.40E-04 | III |
| Citalopram | 11 | 67622 | 0.21 | 1.26(1.05,1.50) | (0.82,1.94) | 45.51 | 0.29 | 1.04E-03 | 1.12E-02 | IV |
| SSRIs (in the first trimester) | 19 | 74191 | 0.63 | 1.26(1.13,1.42) | (0.86,1.87) | 56.06 | 0.16 | 4.29E-15 | 8.23E-05 | III |
| Oral hormone pregnancy tests | 2 | 89 | — | 2.00(0.49,8.11) | — | 24.21 | 1.00 | 2.05E-01 | 3.34E-01 | NS |
| **Mantal disease** | | | | | | | | | | |
| Gestational hypertension | 10 | 109462 | 0.21 | 1.76(1.43,2.16) | (0.84,3.67) | 86.23 | 0.87 | 3.98E-111 | 9.77E-08 | II |
| GDM | 7 | 70344 | 0.70 | 1.63(1.31,2.04) | (0.84,3.17) | 73.06 | 0.45 | 1.26E-44 | 1.29E-05 | III |
| PGDM | 12 | 118826 | 0.10 | 3.39(2.87,4.01) | (1.96,5.88) | 78.11 | 0.87 | 0.00E+00 | 3.74E-46 | II |
| DM | 13 | 120468 | 0.00 | 2.26(1.78,2.87) | (0.90,5.65) | 99.08 | 0.53 | 5.20E-35 | 2.85E-11 | II |

*: Seven factors had only one cohort study including high intake of caffeinated products, solvents exposure, family income, folic acid supplementary, fluconazole (in the first trimester), bupropion, and fever, which did not conduct data quantitative synthesis.

#: Ioannidis’s five-class evidence grade.

**Abbreviation**: CI, Confidence interval; PI, Predictive interval; NS: Not significant associated; IVF: In-vitro-fertilization; ICSI: Intracytoplasmic sperm injection; SSRI, Selective serotonin reuptake inhibitor; DM, maternal diabetes mellitus; PGDM, pregestational diabetes mellitus; GDM, gestational diabetes mellitus; TTTS, twin–twin transfusion syndrome; MC, Monochorionic.


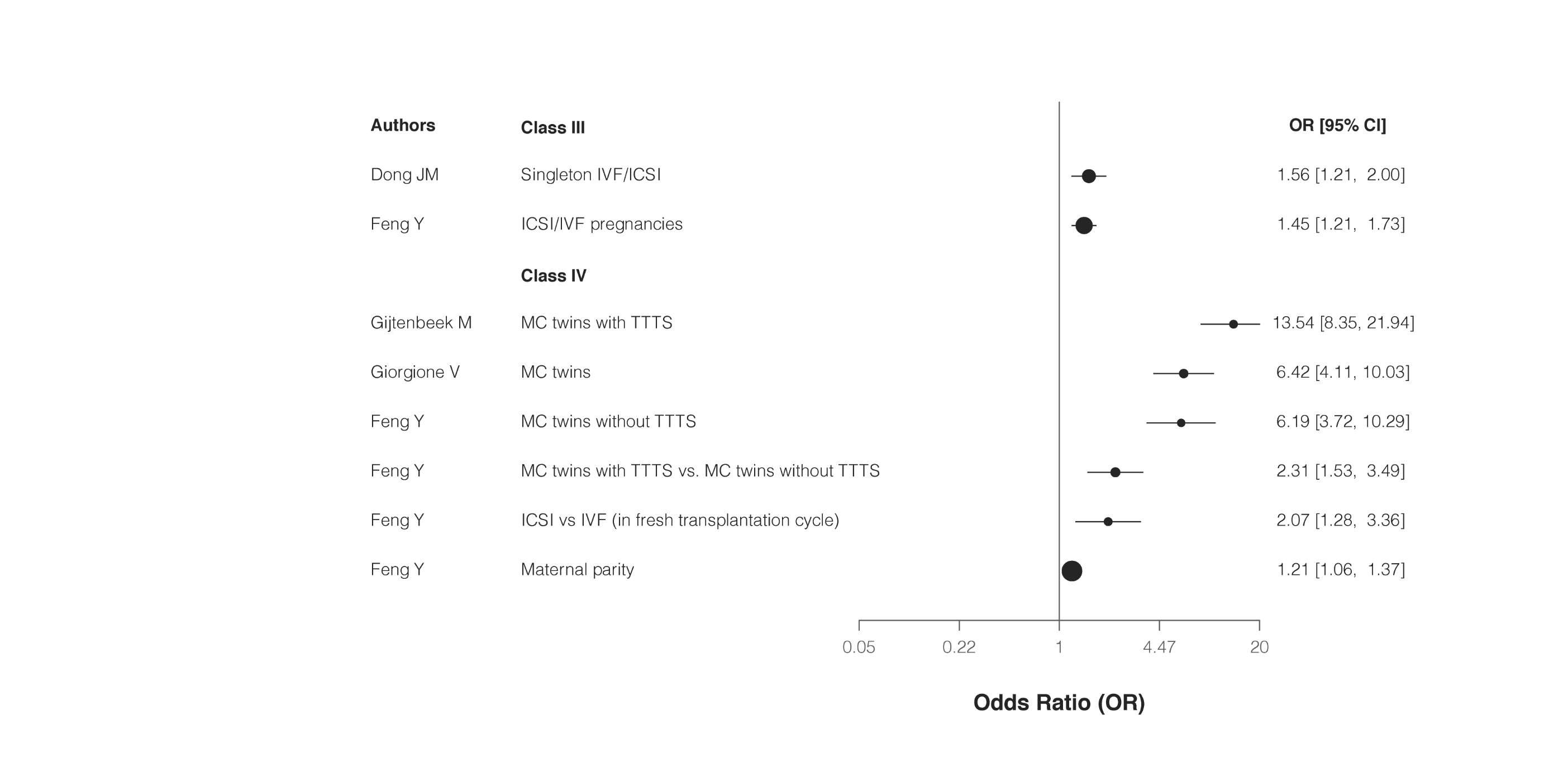


**Supplementary Figure S1** The forest plot of sensitivity analysis for the association between reproductive related and assistive technologies risk factors and CHD

**Abbreviation**: IVF: In-vitro-fertilization; ICSI: Intracytoplasmic sperm injection; TTTS, twin–twin transfusion syndrome; MC, Monochorionic.

**
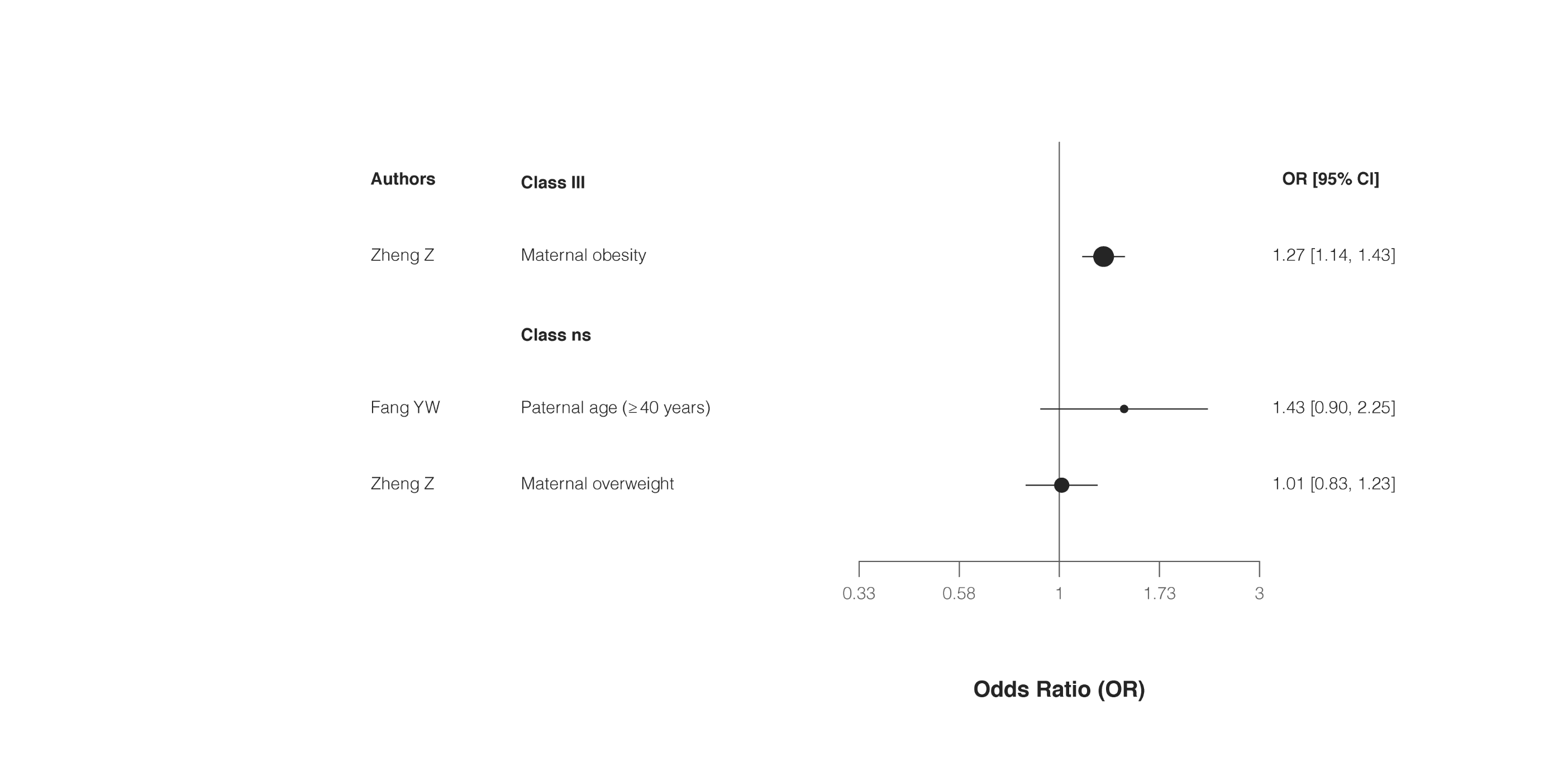
**

**Supplementary Figure S2** The forest plot of sensitivity analysis for the association between parental age and BMI risk factors and CHD


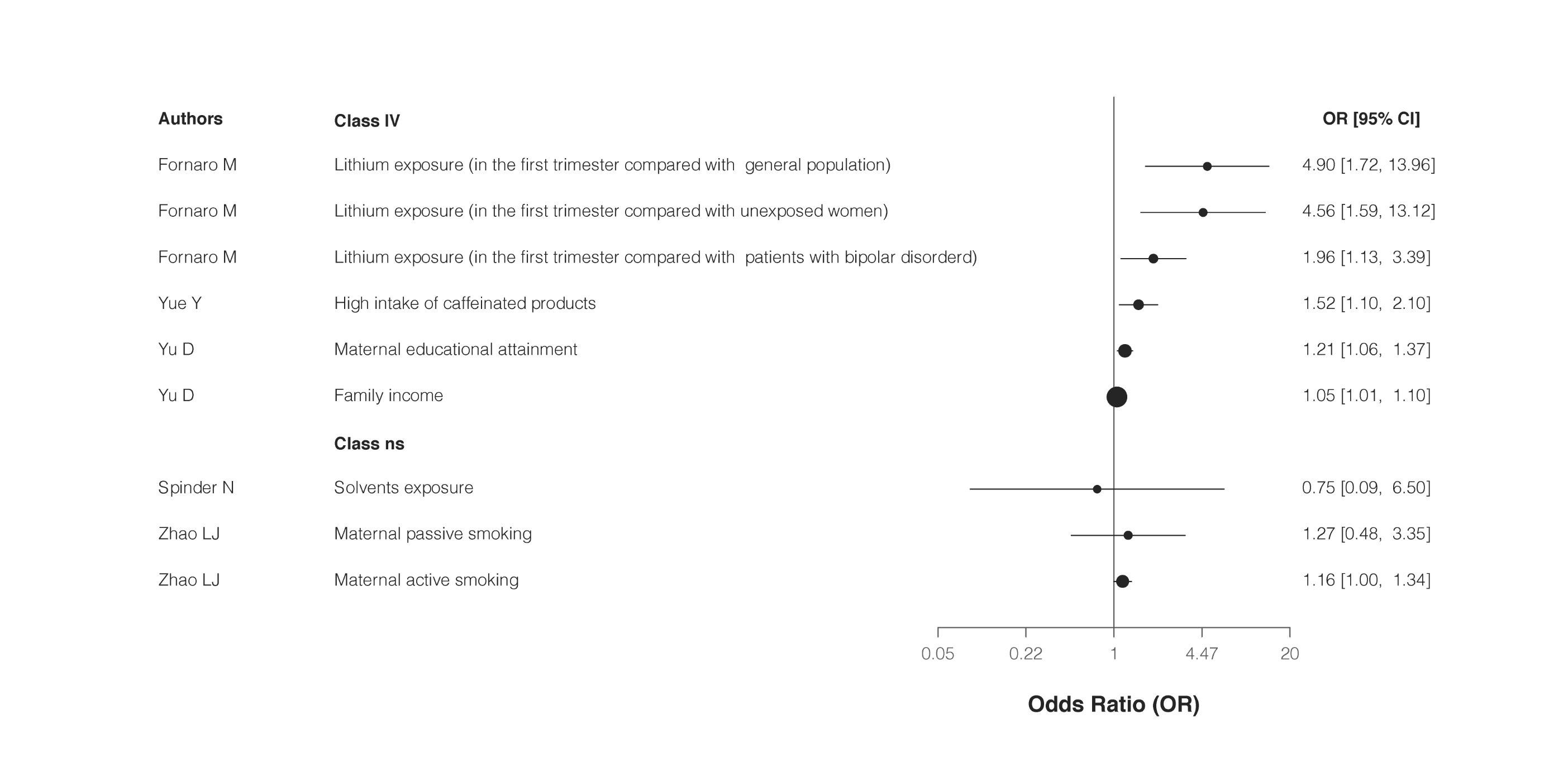


**Supplementary Figure S3** The forest plot of sensitivity analysis for the association between parental life habits, working and dwelling environment risk factors and CHD


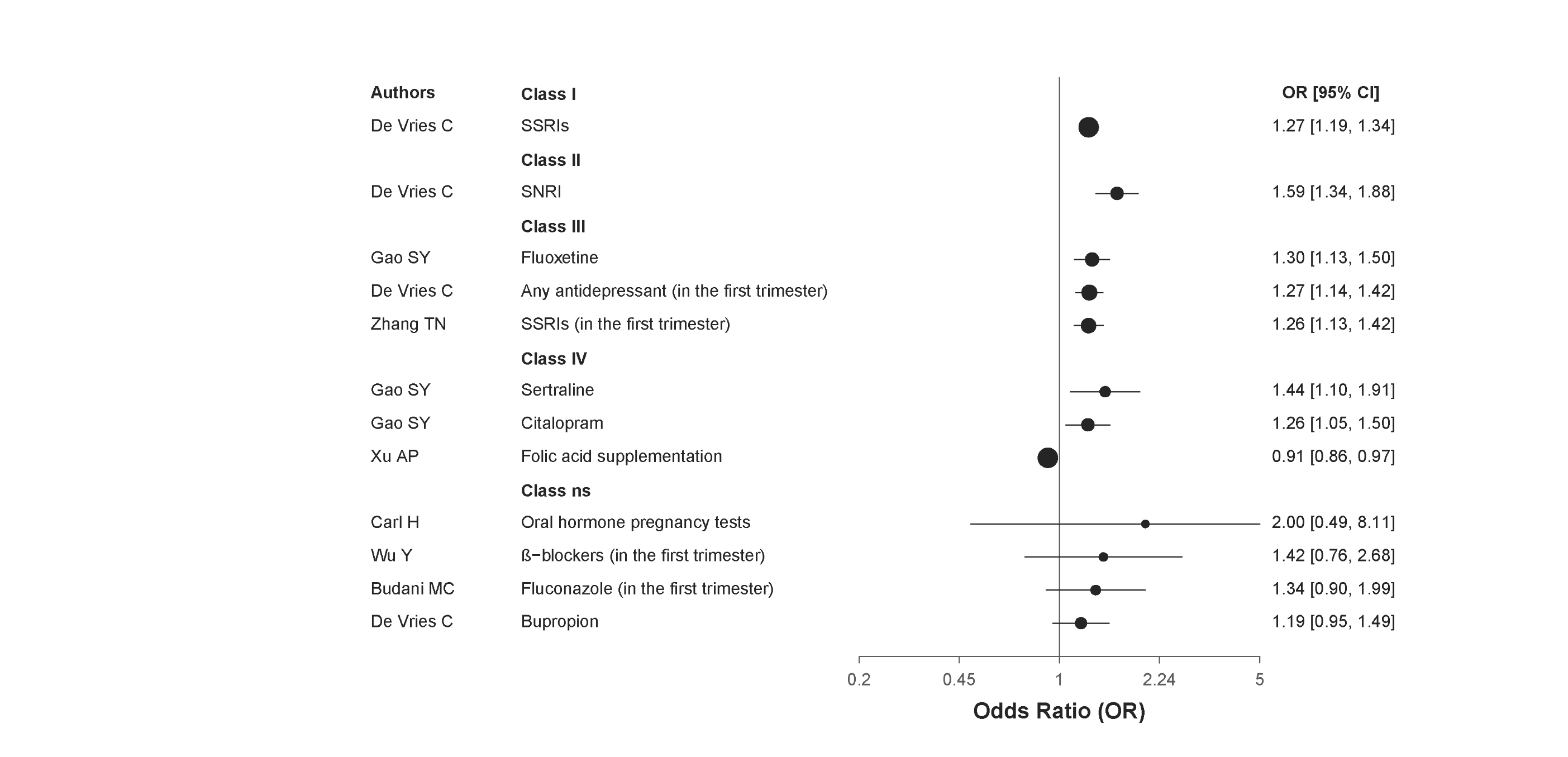


**Supplementary Figure S4** The forest plot of sensitivity analysis for the association between maternal drug exposure risk factors and CHD

**Abbreviation**: SSRI, Selective serotonin reuptake inhibitor; SNRI, serotonin-norepinephrine reuptake inhibitor.

**
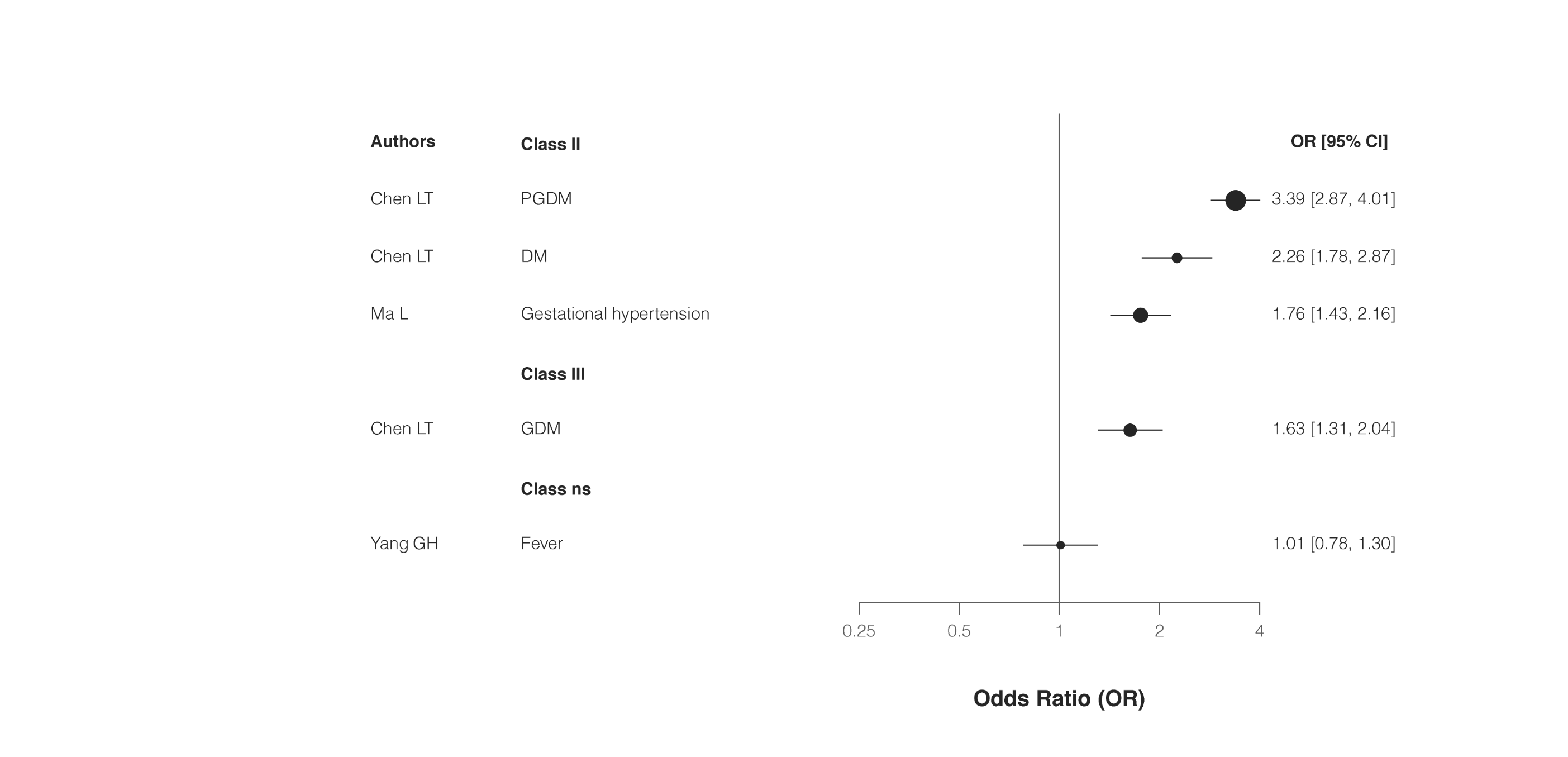
**

**Supplementary Figure S5** The forest plot of sensitivity analysis for the association between maternal disease risk factors and CHD

**Abbreviation**: PGDM, pregestational diabetes mellitus; DM, diabetes mellitus; GDM: gestational diabetes mellitus.
